# Rice iron storage protein ferritin 2 (OsFER2) positively regulates ferroptotic cell death and defense responses against *Magnaporthe oryzae*

**DOI:** 10.3389/fpls.2022.1019669

**Published:** 2022-10-24

**Authors:** Nam Khoa Nguyen, Juan Wang, Dongping Liu, Byung Kook Hwang, Nam-Soo Jwa

**Affiliations:** ^1^ Division of Integrative Bioscience and Biotechnology, College of Life Sciences, Sejong University, Seoul, South Korea; ^2^ Division of Biotechnology, College of Life Sciences and Biotechnology, Korea University, Seoul, South Korea

**Keywords:** cell death, ferritin, ferroptosis, iron, iron-storage protein, *Magnaporthe oryzae*, reactive oxygen species (ROS), rice

## Abstract

Ferritin is a ubiquitous iron storage protein that regulates iron homeostasis and oxidative stress in plants. Iron plays an important role in ferroptotic cell death response of rice (*Oryza sativa*) to *Magnaporthe oryzae* infection. Here, we report that rice ferritin 2, OsFER2, is required for iron- and reactive oxygen species (ROS)-dependent ferroptotic cell death and defense response against the avirulent *M. oryzae* INA168. The full-length ferritin OsFER2 and its transit peptide were localized to the chloroplast, the most Fe-rich organelle for photosynthesis. This suggests that the transit peptide acts as a signal peptide for the rice ferritin OsFER2 to move into chloroplasts. *OsFER2* expression is involved in rice resistance to *M. oryzae* infection. *OsFER2* knock-out in wild-type rice HY did not induce ROS and ferric ion (Fe^3+^) accumulation, lipid peroxidation and hypersensitive response (HR) cell death, and also downregulated the defense-related genes *OsPAL1*, *OsPR1-b*, *OsRbohB*, *OsNADP-ME2-3*, *OsMEK2* and *OsMPK1*, and vacuolar membrane transporter *OsVIT2* expression. *OsFER2* complementation in *ΔOsfer2* knock-out mutants restored ROS and iron accumulation and HR cell death phenotypes during infection. The iron chelator deferoxamine, the lipid-ROS scavenger ferrostatin-1, the actin microfilament polymerization inhibitor cytochalasin E and the redox inhibitor diphenyleneiodonium suppressed ROS and iron accumulation and HR cell death in rice leaf sheaths. However, the small-molecule inducer erastin did not trigger iron-dependent ROS accumulation and HR cell death induction in *ΔOsfer2* mutants. These combined results suggest that *OsFER2* expression positively regulates iron- and ROS-dependent ferroptotic cell death and defense response in rice–*M. oryzae* interactions.

## Introduction

In previous studies, we reported that avirulent *M. oryzae* infection accumulates iron and ROS (H_2_O_2_) in rice leaf sheath cells to trigger the Fenton reaction, which induces lipid peroxidation and consequently initiate ferroptotic cell death in rice (Dangol et al., 2019). Thus, iron may play a critical role in plant immunity, especially in ferroptotic cell death. In cells, iron can exist in the forms of ferrous (Fe^2+^) and ferric (Fe^3+^) ions. In the presence of H_2_O_2_, however, an excess Fe^2+^ inside the cell can damage the cell to produce Fe^3+^ together with a highly toxic •OH through the Fenton reaction (Fenton, 1894; Winterbourn, 1995). Plants have evolved physiological and molecular mechanisms (Aung and Masuda, 2020) and iron regulatory genes (Gross et al., 2003; Quinet et al., 2012; Finatto et al., 2015) for iron uptake, localization, transport and storage, ultimately leading to iron homeostasis in cells. Among the iron regulatory proteins, ferritin is known as a major iron storage protein in plants (Briat et al., 2010).

Ferritin is a large multisubunit protein that stores iron in plants, animals, and bacteria (Ragland et al., 1990). The iron storage protein ferritin has evolved from a common ancestor in plants and animals (Briat and Lobréaux, 1997). The nucleotide sequence of ferritin varies; however, the basic structure is very similar. Basically, ferritin proteins assemble into 24 subunits to form spherical protein cages with large nanocavities, which are capable of enriching thousands of iron atoms (up to ~4500) to form iron protein complexes (Briat et al., 2010; Yang et al., 2015). Each plant ferritin subunit contains two specific domains. First, it is transit peptide domain (TP) with a length of 40–50 residues located in the N-terimal region. It plays a role in subcellular localization of ferritin (Ragland et al., 1990). Second, the extension peptide responsible for protein stability (Briat and Lobréaux, 1997; Briat et al., 2010) belongs to the mature region of the plant ferritin subunit. In the mature region, the extension pepetide is followed by a four-helix bundle (helices A, B, C, and D) and a fifth short helix (E-helix). The E-helix exists around the 4-fold intersubunit symmetry axes of the protein shell to form a hydrophobic pore (Masuda et al., 2001). Plant and animal ferritins share important structural and functional similarities, but little information is known about plant ferritins and their roles in immunity is unclear. In general, the ferritin protein has two functions in cells. First, ferritin can store iron and keep it away from other molecules that may react with it. Second, ferritin can convert iron ions from the reactive form Fe^2+^ to the non-reactive form Fe^3+^, which is safe for cells (Verbon et al., 2017).

Two ferritin genes, *OsFER1* and *OsFER2*, have been identified in the rice genome (Gross et al., 2003). The roles of the rice ferritin genes in plant development and germination against oxidative stress are being studied in rice (Gross et al., 2003; Stein et al., 2009; Strozycki et al., 2010). Most published studies have focused on the role of rice ferritin in defense against iron-mediated oxidative stress. Excessive Fe treatment increased the mRNA and protein levels of rice ferritin in rice cultivars sensitive and tolerant to Fe toxicity, especially higher in resistant cultivars (Silveira et al., 2009). Accumulation of ferritin mRNA in excess iron conditions was significantly higher in copper, herbicide paraquat and sodium nitroprusside (SNP) treatment (Stein et al., 2009). Similarly, the expression of ferritin was significantly higher in roots and shoots by excessive iron treatment (Quinet et al., 2012; Finatto et al., 2015). Thus, OsFER has been described as part of a defense mechanism against Fe toxicity, because the excess Fe is captured by the ferritin protein in a safe and bioavailable form in response to Fe excess (Briat and Lobréaux, 1997). However, the role of rice ferritin in immunity remains unresolved. In potato-*Phytophthora infestans* interactions, ferritin accumulation was detected in leaves and tubers treated with the elicitor eicosapentaenoic acid (Mata et al., 2001). In addition, the *AtFER1* gene was upregulated in *Arabidopsis* during *Erwinia chrysanthemum* infection (Dellagi et al., 2005). Similarly, overproduction of ferritin in transgenic tobacco plants reduced symptoms after viral (tobacco necrosis virus) and fungal (*Alternaria alternata, Botrytis cinerea*) infection (Deák et al., 1999).

In this study, we investigated the roles of *OsFER2* in iron- and ROS-dependent ferroptotic cell death and immune responses in rice (*Oryza sativa*) and blast fungus (*Magnaporthe oryzae*) interactions using T-DNA insertion *ΔOsfer2* knock-out mutants. OsFER2 was localized to the chloroplast, the most Fe-rich organelle for photosynthesis. Avirulent *M. oryzae* INA168 infection significantly induced *OsFER2* expression leading to HR resistant response in rice leaves. However, *OsFER2* knock-out distinctly inhibited iron and ROS accumulation, lipid peroxidation and HR cell death in *ΔOsfer2* mutant cells, which ultimately induced susceptible responses to avirulent *M. oryzae* INA168 infection. A *OsFER2* complementation test verified that *OsFER2* is the causal gene for *ΔOsfer2* mutant phenotypes. Collectively, these results suggest that *OsFER2* is involved in iron- and ROS-dependent ferroptotic cell death and immune response in rice during avirulent *M. oryzae* infection.

## Materials and methods

### Plant materials and growth conditions

All the experiments in this study were carried out using rice (*Oryza sativa*) cultivar Hwayeongbye (HY) (Dangol et al., 2019), *ΔOsfer2* knock-out mutants and *OsFER2* complementation lines. Wild-type rice HY seeds were obtained from the National Institute of Crop Science (http://www.nics.go.kr) in Korea. *ΔOsfer2* T-DNA insertion mutant seeds were provided by the Rice Functional Genomic Express Database managed by the Salk Institute (http://signal.salk.edu./cgi-bin/RiceGE) (Jeon et al., 2000). Plants were raised in growth chambers under the controlled conditions of 16 h light-8 h dark at 28°C.

#### Isolation and Identification of Rice Ferritins *OsFER1* and *OsFER2*

The coding sequence information of OsFER1 (LOC_11gOs01530) and OsFER2 (LOC_12gOs01530) was provided by the Rice Genome Annotation Project (http://rice.uga.edu/). The two rice ferritin cDNA genes were amplified from a rice cDNA library using the gene-specific primers with attB1 and attB2 sites. The PCR products were purified and cloned onto the entry vector pDONR201 by Gateway™ BP Clonase™ II Enzyme (Invitrogen). The full-length coding sequences were identified by DNA sequencing (Macrogen, Seoul, Korea).

### Fungal cultures and growth conditions

*Magnaporthe oryzae* strains PO6-6 and INA168 that were virulent (compatible) and avirulent (incompatible) to the rice cultivar HY, respectively, were provided by the Center for Fungal Genetic Resources, Seoul National University (http://genebank.snu.ac.kr). *M. oryzae* strains were cultured on the medium (20 g rice bran, 20 g glucose and 20 g agar in 1 L H_2_O) at 25°C. *M. oryzae* INA168 was grown in the continuous light condition for 3 weeks. *M. oryzae* PO6-6 was also grown in dark condition for 2 weeks, followed by 1 week in light after removing all aerial mycelia from the plates.

### Fungal inoculation and infection evaluation

Conidia of *M. oryzae* were harvested from the sporulated plates using sterilized tap water with 0.025% (v/v) Tween 20 (Sigma-Aldrich). The conidial numbers in suspensions were counted using a hemacytometer and adjusted to 4x10^5^ conidia/mL. The harvested conidial suspension (4x10^5^ conidia/mL) was spray-inoculated over 3-week-old rice plants. The seedling plants were keep at 28°C in dark condition for 24 h, followed by 4 day-incubation in normal growth condition (16 h/8 h, dark/light). The infected seedling leaves were photographed 5 days after inoculation with *M. oryzae.* The virulence levels of *M. oryzae* strains used were evaluated on the leaves of rice cultiver HY, as described in previous study (Dangol et al., 2019).

The rice leaf sheaths from 3-5-week-old rice plants were used for inoculation with *M. oryzae*, as described previously (Kankanala et al., 2007; Dangol et al., 2019). Rice leaf sheaths were cut into 4-5 cm in length and inoculated with the freshly prepared conidial suspension (4x10^5^ conidia/mL) of *M. oryzae*. The inoculated leaf sheaths were then kept in a moistened box with 100% relative humidity in the dark condition at 25°C. At the different time points after inoculation, the middle thin epidermal layers were excised from the leaf sheaths and observed under the microscopes. The infected cells were divided into the two infection phenotypes: cells with visible invasive hyphae (IH) and hypersensitive response (HR) cell death. The number of cells of each phenotype are counted four times from different leaf sheath samples in three independent experiments.

### Identification of T-DNA insertion in *ΔOsfer2* mutants

Genomic DNA of rice mutant seeds was isolated by cetyltrimethyl ammonium bromied (CTAB) buffer for PCR. T-DNA insertion mutant (*ΔOsfer2*) seeds from RiceGE (Jeon et al., 2000) were screened by PCR using the gene primers (LP and RP) and the vector primers (LP and RB). The presence of hygromycin resistance gene in *ΔOsfer2* mutants was confirmed by partial hygromycin primers (HPT F/R). To verify the rice HY cultivar background of the *ΔOsfer2* mutant seeds, a pair of primers Pib F/R was used to detect the blast resistance (R) gene Pib (Cho et al., 2007). The amplification fragments were sequenced to determine the flanking sequences.

### Real-time RT-PCR analyses

Total RNA of leaf sheath tissues at the 3-week-old leaf stage was extracted by TRIzol reagent (Invitrogen). cDNA was synthesized by the SuperScript III Reverse Transcriptase (Invitrogen). qRT-PCR was performed using TOPreal™ qPCR 2x PreMIX (SYBR Green with low ROX; Enzynomics, Daejeon, Korea) by Mx3005P qPCR System (Agilent Technologies). Relative gene expression levels were determined using rice 18S ribosomal RNA (*18S rRNA*) or rice *OsUbiquitin* as internal standard genes.

### Subcellular localization of OsFER2 and its domains in *N. benthamiana* leaves

The full-length *OsFER2* cDNA was cloned into the entry vector pDONR201. Transit peptide and mature regions were isolated from the full length *OsFER2* cDNA in pDONR201. The start codon ATG is included in the N-terimal sequence of mature region clones. The PCR products were then cloned into pDONR201 in the same way as full length sequences. The clones in pDONR201 were recombined into the Gateway cloning compatible binary vector PGWB552 (N-terminal fusion with G3GFP) using Gateway™ LR Clonase™ II Enzyme (Invitrogen). The rice chloroplast precursor protein (OsERD1) fused with mRFP in the N-terminal region was used as a chloroplast marker, as described previously (Dangol et al., 2017). All the primer information is listed in **Supplementary Table 1**.

The binary plasmid pGWB552 containing the *OsFER2* full length and domain regions was introduced into *A. tumefaciens* GV3101. The *Agrobacterium* infiltration protocol was described previously with a slight modification (Sainsbury and Lomonossoff, 2008). *Agrobacterium* colonies grown on the LB plate containing 100 μg/ml spectinomycin was cultured in the LB-spectinomycin at 30° C for 2 days in a horizontal shaker. The cells were suspended in the infiltration buffer (10 mM 2-morpholinoethanesulfonic acid (MES) pH 5.6, 10 mM MgCl_2_, 150 mM acetosyringone) and adjusted to an OD_600_ = 0.2. The *Agrobacterial* suspension was incubated for 2 h at room temperature and then infiltrated to the abaxial *Nicotiana benthamiana* leaves. The agro-infiltrated plants were kept at 25° C in dark for 24 h, followed by light condition for 24 h. After incubation for 48 h, thin epidermal layers were isolated from leaves and stained for 5 min in 4’,6-diamidino-2-phenylindole (DAPI, 5 µg/mL in 1x PBS), followed by 5-min-washing with 1x PBS solution. The subcellular localization of full length and domain regions of OsFER2 in *N. benthamiana* was observed under a fluorescence microscope (Olympus, Japan).

### Chemical treatment

The actin microfilament polymerization inhibitor cytochalasin E (Cyt E) and the iron chelator deferoxamine (DFO) were used in this study to suppress ferroptosis by reducing availability of iron (Dangol et al., 2019). For Cyt E treatment, 10 µg/mL Cyt E solution was treated onto the infected rice leaf sheaths at 24 hpi, followed by 24 hr-incubation in the dark at 25°C. The rice leaf sheaths were treated with 3 mM DFO at 42 h after inoculation with *M. oryzae*, following by 6 hr-incubation in the same condition. The NADPH oxidase inhibitor diphenyleneiodonium (DPI) was used for suppression of ferroptotic cell death (Dixon et al., 2012; Dangol et al., 2019). The DPI chemical was mixed with the conidial suspension (4x10^5^ conidia/mL) of *M. oryzae* to the final concentration 5 µM DPI. The conidial suspension of *M. oryzae* in 5 µM DPI was inoculated onto rice leaf sheaths (5–7 cm in length), followed by 48 hr-incubation in the dark at 25°C. The small-molecule ferrostatin-1 (Fer-1), an lipid ROS scavenger, was used for blocking lipid peroxidation in ferroptosis (Dixon et al., 2012; Stockwell et al., 2017). Infected rice leaf sheaths at 24 hpi was treated with 10 µM Fer-1 solution, followed by 24-h incubation in the same condition.

Erastin is known as a small-molecule inducer of ferroptotic cell death in mammalian cells (Dixon et al., 2012). Erastin was mixed with the freshly harvested *M. oryzae* conidial suspension (4x10^5^ conidia/mL) to the concentration of 10 µM. The *M. oryzae* conidial suspension in 10 µM erastin was inoculated onto rice leaf sheaths, followed by 48 hr-incubation at 25°C in dark condition. As controls against all chemical treatments, mock(water) treatments were performed on rice leaf sheaths of rice HY and *ΔOsfer2* mutant plants at the same time. After incubation with treated chemicals, the thin epidermal layers of rice leave sheaths infected with *M. oryzae* were observed under the microscopes.

### Cellular ROS detection by CM-H_2_DCFDA and DAB staining

CM-H_2_DCFDA [5-(and-6)-chloromethyl-2’,7’-dichlorodihydrofluorescein diacetate, acetyl ester] (Invitrogen) and DAB (3,30-diaminobenzidine) (Sigma-Aldrich) were used to detect reactive oxygen species (ROS) in rice leaf sheath cells, as described previously (Dangol et al., 2019; Dangol et al., 2021). CM-H_2_DCFDA staining was use for monitoring ROS formation and localization in living cells (Kristiansen et al., 2009). Briefly, thin epidermal layers of rice leaf sheaths infected with *M. oryzae* were cut into 2-3 cm length, followed by incubation in water for 5 min at 4° C. The epidermal layers of rice leaf sheaths were stained with 2 µM CM-H_2_DCFDA in 1x phosphate-buffered saline (PBS) buffer in the dark condition for 30 min on a horizontal shaker, followed by 3 time-washings with 1x PBS buffer every 5 min. ROS localization inside the epidermal sheath cells was visualized under a fluorescence microscope. DAB reacts with ROS in the presence of peroxidase to form a deep brown polymerization product (Thordal‐Christensen et al., 1997). For DAB staining, the epidermal layers were stained in 1 mg mL^−1^ DAB for 1 h, followed by detaining overnight in the solution ethanol:acetic acid:glycerol (3:1:1, v/v/v). The DAB-stained cells were observed under bright-fields in microscope.

### Ferric ion detection by prussian blue staining

Prussian blue staining solution [7% (w/v) potassium ferrocyanide and 2% (v/v) hydrochloric acid (1:1, v/v)] was used to detect ferric ion (Fe^3+^) in rice leaf sheath cells, as described previously (Dangol et al., 2019). The thin epidermal layers of rice leaf sheaths infected with *M. oryzae* were cut into 2-3 cm length and incubated in the staining solution for 15 h at room temperature in a gentle shaking mode. Fe^3+^ inside the cell reacts with ferrocyanides to form ferric ferrocyanides (Prussian blue, a bright blue pigment) (Liu et al., 2007; Dangol et al., 2019). The Prussian blue-stained cells were observed under a bright-field microscope and classified into the two phenotypes: unstained cells that contain invasive hyphae (IH) but are weakly or not Prussian blue-stained; and stained cells, strongly Prussian blue-stained cells with only a few poor hyphae.

### Chemiluminescence and malondialdehyde assays for ROS measurement

The thin epidermal layer of rice sheaths infected with *M. oryzae* was cut into small pieces (0.5 cm length), followed by 5-min incubation in water to remove wound-induced ROS. In the black 96-well plates, a small piece of rice sheath was incubated for 5 min in the reaction solution [30 µL of luminol (Bio-Rad), 1 µL of horseradish peroxidase (Jackson Immunoresearch), and 69 µL of Milli-Q water]. The chemiluminescence (RLU) (ROS levels) were measured by the GloMax 96 Microplate Luminometer (Promega, Seoul, Korea).

Malondialdehyde (MDA) assay was used to quantify lipid peroxidation levels in rice leaf sheath tissues, as described previously (Dangol et al., 2019; Dangol et al., 2021). Briefly, the rice sheath was grinded in liquid nitrogen into fine powder, followed by mixing the tissue powder in the reaction solution [0.5% (w/v) thiobarbituric acid, 20% (v/v) trichloroacetic acid (TCA), and 0.25 mL 175 mM NaCl in 2 mL of 50 mM Tris-Cl, pH 8.0]. The mixtures were boiled for 5 min in a hot water bath, followed by 5-min-cooling in ice. After centrifuging the samples at 14,000 *g*, the absorbance of the resultant supernatants was measured at optical density (OD): 450, 532, and 600 nm. MDA concentrations were calculated by the equation: C=6.45 × (OD_532_-OD_600_) - (0.56 × OD_450_) (Dangol et al., 2019; Dangol et al., 2021).

### Complementation of *OsFER2* in *ΔOsfer2* mutants

*OsFER2* coding sequence was amplified from rice cDNA library and cloned into the entry vector pDONR201 using the primers (**Supplementary Table 1**). The entry clones were then recombined into the Gateway binary vector pB2GW7. The destination vector contains cauliflower mosaic virus (CaMV) 35S promoter in the upstream of inserted *OsFER2* clone and streptomycin and/or spectinomycin resistance gene for plasmid selection and the BAR gene for plant selection (Karimi et al., 2002). The constructed CaMV 35S:*OsFER2* was transformed into *Agrobacterium tumefaciens* strain LBA4404, which was then delivered to *ΔOsfer2* #3 rice callus, as described previously with slight modification (Hiei et al., 1994; Wu et al., 2003).

Briefly, the *OsFER2-*complemented rice plants were created from the *ΔOsfer2* calli, which were induced on the 2N6 medium for 3-4 weeks. The intact *ΔOsfer2* calli and *A. tumefaciens* carrying the construct CaMV 35S:*OsFER2* were co-cultured for 3 days in dark condition. The agro-infected and transformed calli was then induced in dark condition at 25° C for 1 week on the 2N6 medium containing cefatoxine (250 mg/L), and for 3 weeks on the 2N6 medium containing cefatoxine (250 mg/L) and 2 mg/L DL-phosphinothricin (PPT) (Duchefa). The induced calli were regenerated in light condition at 25° C on the 2N6 medium containing cefatoxine (250 mg/L), kinetin (1 mg/L), NAA (2 mg/L) and PPT (4 mg/L). After 3 week-culture in the regeneration media, the calli was transferred to ½ MS medium for rooting and shooting. When rice plants emerged from the calli, they were transferred to ½ MS medium plates and tall bottles and then moved to the soil.

### Microscopy

The images of infected cells were captured by ZEISS Axioplan 2 imaging microscope using 40x oil-immersion objective lenses in bright field. Excitation (450‒490 nm) and emission (515‒565 nm) of green fluorescent filters were used to visualize fluorescence signals in CM-H_2_DCFDA.

### Accession numbers

Sequence data from this article were collected from the Rice Genome Project website (http://rice.uga.edu/): *OsFER1* (Os11g01530), *OsFER2* (Os12g01530), Os-*NADP-ME2-3* (Os01g52500), *OsMPK1* (Os06g06090), *OsMPK6* (Os10g38950), *OsWRKY90* (Os09g30400), *OsRbohB* (Os01g25820), *OsPR1-b* (Os01g28450), *OsAPX1* (Os03g17690), *OsAPX2* (Os07g49400), *OsPAL1* (Os04g43760), *OsUbiquitin* (Os06g46770), *OsERD1* (Os02g32520) and the National Center for Biotechology Information: *TritaFer1* (AY864925), *TritaFer2* (EU143671), *ZmFer1* (X61391), *ZmFer2* (X61392), *SFerH1* (M64337), *SFerH2* (AB062754), *SFerH3* (AB062755), *SFerH4* (AB062755), *AtFer1* (AT5G01600), *AtFer2* (AT3G11050), *AtFer3* (AT3G56090), *AtFer4* (AT2G40300), *NtFer1* (AY083924), and *NtFer2* (AY141105).

## Results

### Identification of Ferritin-genes in *Oryza sativa* genome

**Figure 1:**
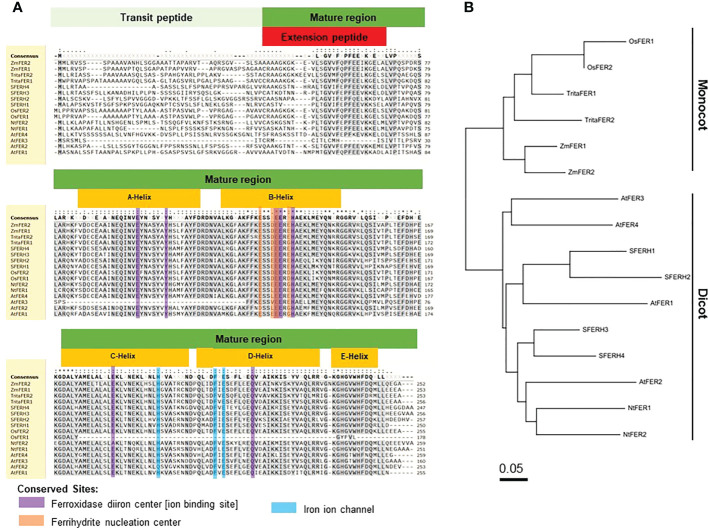
Amino acid sequence alignment and phylogenetic tree of rice (*Oryza sativa)* ferritins with other plant ferritins. **(A)** Amino acid sequence alignment and **(B)** phylogenetic tree of ferritins in *Oryza sativa*, *Triticum aestivum*, *Zea mays*, *Glycine max*, *Arabidopsis thaliana*, and *Nicotiana benthaminana.* The phylogenetic tree of ferritins was constructed using MEGA X: Molecular Evolutionary Genetics Analysis across computing platforms (Kumar et al., 2018). Amino acid sequence alignment is presented by SnapGene Viewer version 5.2.4. Residues that are conserved across at least 70% sequences were highlighted in grey. Different symbols indicate identical amino acid residues: conserved sequence (*), conservative mutations (:), semi-conservative mutations (.), and non-conservative mutations ( ). The global consensus sequence is shown on top of the alignment; gaps are represented by (**-**) and ambiguous symbols are represented by (X). Accession numbers: OsFER1 (Os11g01530), OsFER2 (Os12g01530), TritaFER1 (AY864925), TritaFER2 (EU143671), ZmFER1 (X61391), ZmFER2 (X61392), SFERH1 (M64337), SFERH2 (AB062754), SFERH3 (AB062755), SFERH4 (AB062755), AtFER1 (AT5G01600), AtFER2 (AT3G11050), AtFER3 (AT3G56090), AtFER4 (AT2G40300), NtFER1 (AY083924), and NtFER2 (AY141105).

Based on the information from the Rice Genome Annotation Project, rice (*Oryza sativa*) ferritin *OsFER1* (LOC_11gOs01530) has two different alternative splicing forms with the same coding regions. However, there are three different alternative splicing forms in rice ferritin *OsFER2* (LOC_12gOs01530), which are slightly different in size but share the same N-and C-terminal region sequences (**Supplementary Figures 1A, B** **)**. We amplified the alternative splicing forms from the rice cDNA library using the PCR and detected only the band size of the form 1 or 2 of *OsFER2* (**Supplementary Figures 1A, C** **)**. The amplified *OsFER* cDNAs of rice from the rice cDNA library were cloned onto the vector pDONR201 and have been sequenced by a pDONR201 forward primer (**Supplementary Figures 1**–**3**). *OsFER1* sequence was completely matched with the sequence from the database of Rice Genome Annotation Project (**Supplementary Figure 2**). However, the *OsFER*2 sequence was highly matched with the alternative splicing form 2, except the three base pairs in the nucleotide sequence that is different in the amino acid number 145 (from Methionine to Isoleucine) (**Supplementary Figures 1**, **3** **;** **Figure 1A**). These data indicate that there are *OsFER1* and *OsFER2* encoding different ferritins in rice (*Oryza sativa*). The corresponding coding sequence of *OsFER1* and abundant form of *OsFER2* were identified in this study.

### Structures and amino acid sequences of rice ferritin and other plant ferritin proteins

Ferritin is a ubiquitous iron storage protein, which is found in all living kingdoms (Briat et al., 2010). Rice ferritin identities are very similar to those of other representative plant specices (**Figures 1A, B** **)**. In the monocot ferritin group, rice (*Oryza sativa*) shares at least 72.9% and 72.5% sequence identity to wheat (*Triticum aestivum*) and maize (*Zea mays*), respectively. Rice ferritin proteins share more than 58.8% sequence identity to those of *Nicotiana tabacum. Arabidopsis thaliana* has four different ferritin proteins which share at least 46% sequence identity to rice ferritin subunit proteins. Compared to soybean (*Glycine max*), the sequence identities are 50.9-58.9% for OsFER1 and 58.4-66.0% for OsFER2 (**Supplementary Figure 4**). Most plant ferritin proteins conserve the amino acids essential for possible functions of the ferritin proteins, such as the ferroxidase diiron center, ferrihydrite nucleation center, and iron ion channel (**Figure 1A**). This indicates that the functions associated with these plant-specific domains are highly conserved in all ferritins found in plants.

The two rice ferritin proteins, OsFER1 and OsFER2, share about 93.3% sequence homology (**Supplementary Figure 4**). Rice transit peptides are highly conserved in OsFER1 and OsFER2; however, mature regions have evolved diversely. Compared to OsFER2, OsFER1 lacks C-, D- and partial E-helix that have some functional amino acids. Since C- and D-helix of OsFER2 contain some functional amino acids that are not present in OsFER1, OsFER2 is expected to play a major role in the storage and release of iron atoms in native ferritin cages (**Figure 1A** **)**. Hence, *OsFER2* was selected for use in this study.

### OsFER2 and its transit peptide domain are localized to the chloroplast

**Figure 2:**
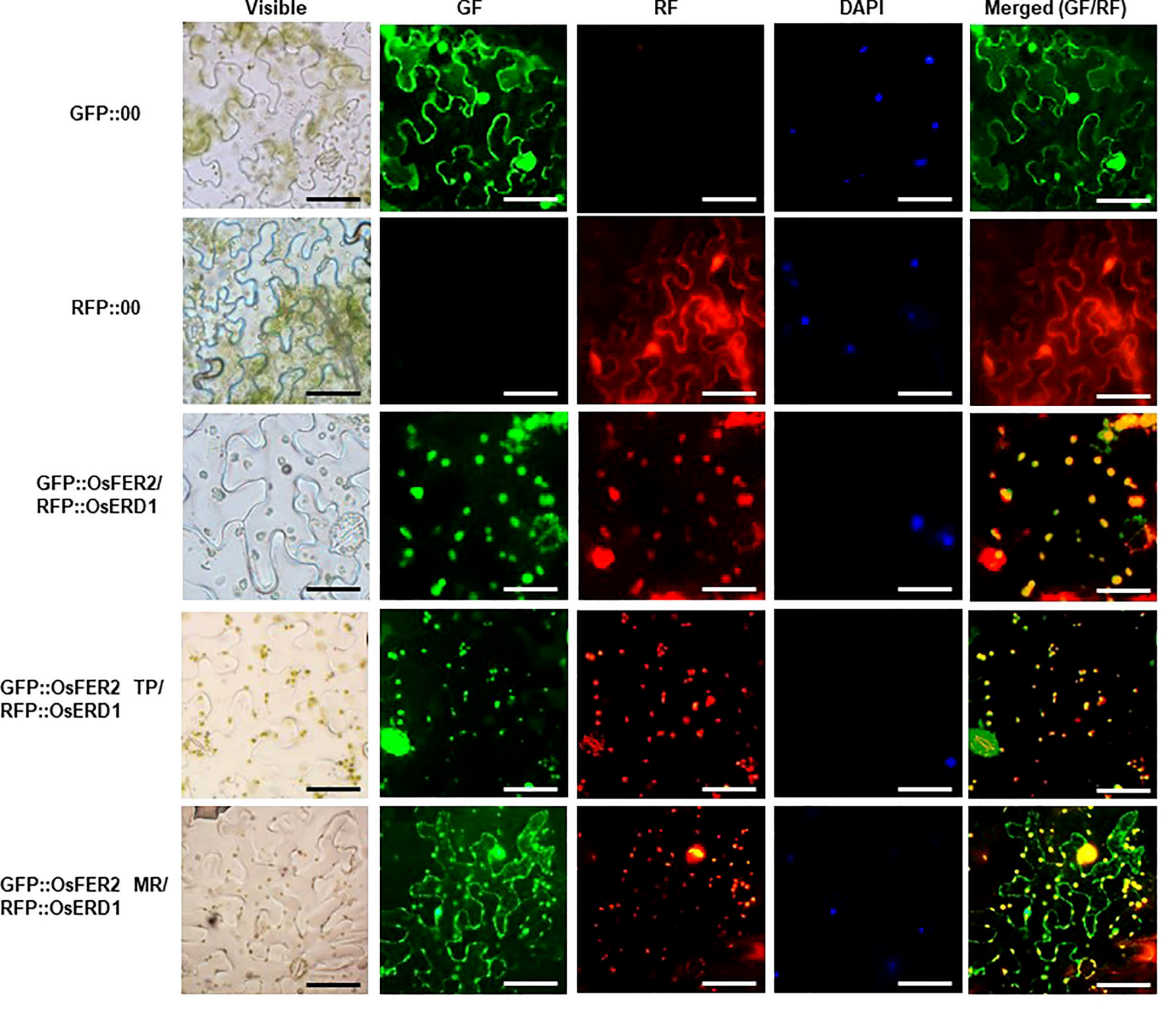
Subcellular localization of OsFER2 and its domains at 48 h after agroinfiltration into *Nicotiana benthamiana* leaves. 4’,6-diamidino-2-phenylindole (DAPI) staining was used to visualize nuclei in *N. benthamiana* epidermal cells. RFP::OsERD2 was used as a chloroplast marker. Images were taken with a fluorescence microscope using bright field (visible), GF (green fluorescence), RF (red fluorescence) and DAPI filters. GFP, green fluorescent protein; RFP, red fluorescent protein; TP, transit peptide; MR, mature region. Scale bar=50 µm.

Ferritin is a major iron storage protein in chloroplasts (Merchant and Dreyfuss, 1998; Buckhout et al., 2009; Briat et al., 2010; Waters et al., 2012). Plant ferritin is localized to the chloroplast, a unique organelle in plants. To understand the biological function of OsFER2 in plant cells, we investigated the subcellular localization of OsFER2 and its domains in *N. benthamiana* cells (**Figure 2**). *OsFER2* and its domains were fused to N-terminal GFP-tagged vector pGWB552 and then transiently expressed in *N. benthamiana* cells. RFP::OsERD2 was used as a chloroplast marker in this study. The nuclei inside cells were counterstained with DAPI. The control GFP- or RFP-constructs (GFP::00 or RFP::00) were ubiquitously detected in the cytoplasm of *N. benthamiana* cells. However, GFP::OsFER2 was mainly localized to the chloroplast (**Figure 2**). Similarly, the OsFER2 transit peptide (GFP::OsFER2/TP) also localized in the chloroplast, as observed overlapping with the chloroplast marker OsERD1 localization. In contrast, the localization of the OsFER2 mature region was ubiquitous in the cytoplasm, chloroplast and nucleus. Collectively, the subcellular localization data provide clues that chloroplasts are the organelle of action of OsFER2 in cells. Notably, the OsFER2 transit peptides may function as a chloroplast-targeting domain.

### *OsFER2* expression is involved in rice resistance during avirulent *M. oryzae* infection

**Figure 3:**
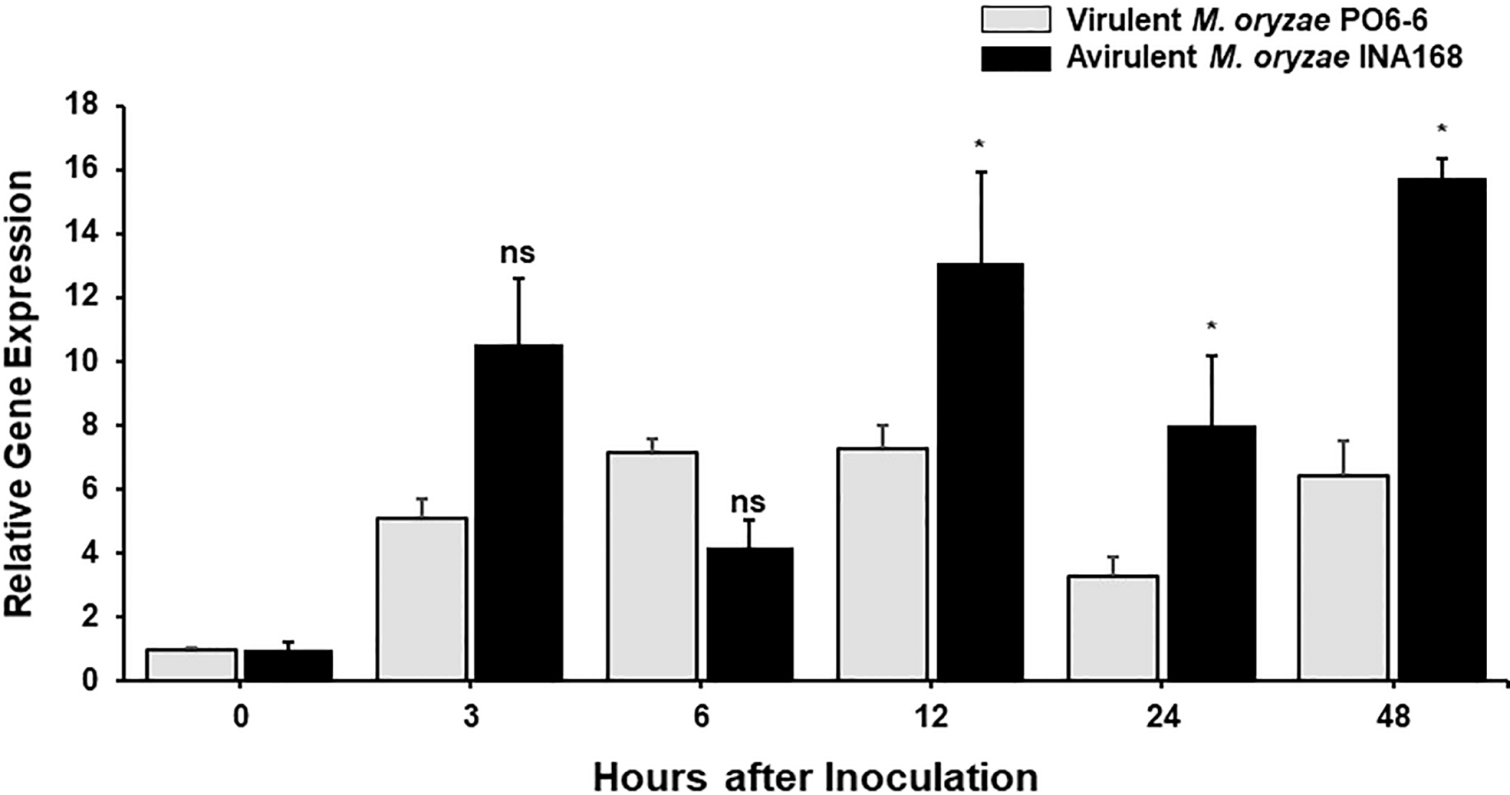
*OsFER2* expression levels in leaf sheaths of rice HY in the compatible and incompatible interactions between rice and *Magnaporthe oryzae*. Rice leaf sheaths were inoculated with *M. oryzae* PO6-6 (virulent) and INA168 (avirulent). *OsFER2* expression was analyzed by quantitative RT-PCR. Relative gene expression of *OsFER2* at time points after inoculation were calculated by normalizing with respect to the expression of the internal control *OsUbiquitin*. The data represent the means ± SDs from the three independent experiments. Asterisks above the columns indicate significant differences as analyzed by Student’s *t*-test (*P<0.05). ns, not significant.

We investigated whether *OsFER2* expression is involved in rice resistance during *M. oryzae* infection. *OsFER2* expression levels in leaf sheath tissues of rice HY plants at the time of initial infection were analyzed by real-time quantitative RT-PCR (**Figure 3**). Both *M. oryzae* PO6-6 (virulent) and INA168 (avirulent) infections significantly induced *OsFER2* expression in rice leaf sheaths at the early infection stage up to 48 hpi, compared to uninoculated healthy ones. Induction of *OsFER2* expression by *M. oryzae* infection was similar in compatible and incompatible interactions between rice and *M. oryzae* by 6 hpi. However, avirulent *M. oryzae* INA168 infection induced distinctly more *OsFER2* expression in rice leaf sheaths at 12-48 hpi than virulent *M. oryzae* PO6-6 infection. These *OsFER2* expression patterns in rice indicate that the *OsFER2* gene is involved in rice blast disease and immunity at the early infection stage during *M. oryzae* infection.

### Identification and disease-susceptible phenotypes of *ΔOsfer2* mutant plants

**Figure 4:**
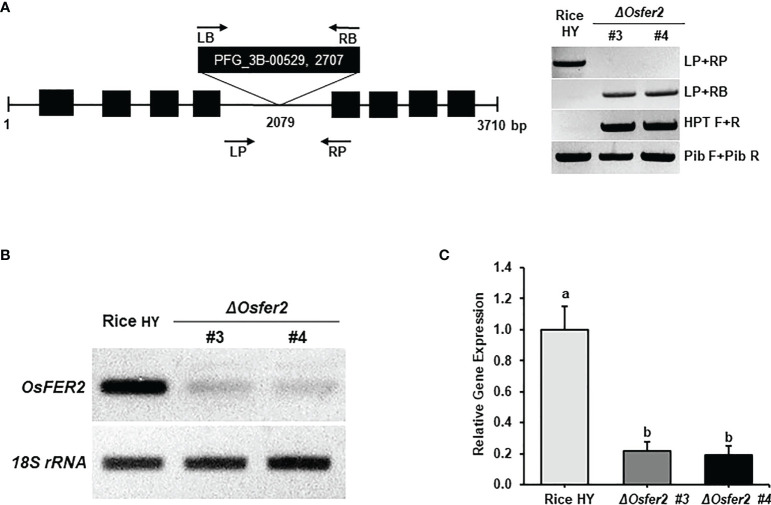
Genotyping and transcriptional analyses of *ΔOsfer2* knock-out lines. **(A)** Genotyping of *ΔOsfer2* plants. The schematic diagram illustrates the T-DNA insertion site in the *OsFER2* gene. The black boxes and lines show exons and introns, respectively.The arrows indicate the orientation of T-DNA insertions from the left border (LB) to the right border (RB).The T-DNA insertion *ΔOsfer2* mutant plants were detected using the gene primers (LP and RP) and the vector primers (LB and RB). The insertion site (2079 bp) of T-DNA was detected by sequencing. Hygromycin (HPT) primers (HPT F/R) were used for dectecting the hygromycin selection gene in the mutants. Pib (rice resistance gene) primers (Pib F/R) were used for detecting the Pib resistance gene existed in rice HY cultivar (Cho et al., 2007). LP, gene left primer; RP, gene right primer; LB, T-DNA left border; RB, T-DNA right border. **(B, C)** Transcriptional analyses of *OsFER2* expression in rice HY and *ΔOsfer2* knock-out lines by RT-PCR and qRT-PCR. Relative gene expression of *OsFER2* in rice leaf sheaths was calculated by normalizing with respect to the expression of the internal control *18S rRNA.* The data represent the means ± SDs from the three independent experiments. Different letters above the bars indicate significantly different means (*P*<0.05), as analyzed by one-way ANOVA analysis.

*ΔOsfer2* knock-out mutant lines was generated from rice cultivar HY by T-DNA insertion mutagenesis. The *OsFER2* genomic DNA contains eight exons and seven introns (**Figure 4A**). T-DNA insertion in *OsFER2* was detected by the primer set LP+RB. The presence of T-DNA insertion inside *ΔOsfer2* mutants was reconfirmed by detecting the hygromycin section gene using HPT primers (HPT F/R). No T-DNA insertion in rice HY was confirmed by the absence of amplification in PCR using the gene primer set (LP+RP). The amplification by the gene specific primer and T-DNA primer was sequenced to point out the exact insertion site (2079 bp). The T-DNA was detected in the fourth intron of *OsFER2* genomic DNA. *ΔOsfer2 #3* and *#4* were identified as T-DNA insertion homozygous plants using the primer set LP+RB (**Figure 4A**). *OsFER2* expression levels were determined by RT-PCR and qRT-PCR (**Figures 4B, C** **)**. *OsFER2* expression was completely suppressed in *ΔOsfer2 #3* and *#4* lines, compared to wild-type rice HY.

**Figure 5:**
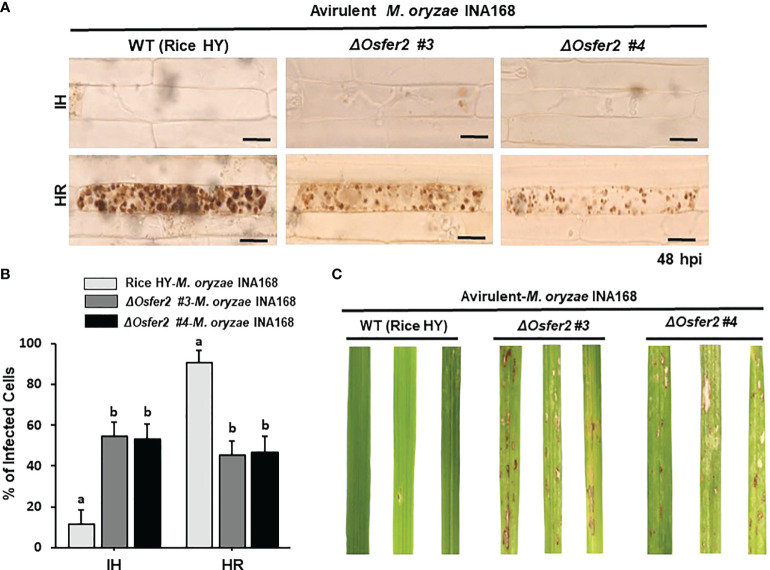
*OsFER2* knock-out in the resistant cultivar HY suppresses resistant phenotypes in *ΔOsfer2* knock-out mutants against avirulent *Magnaporthe oryzae* INA168 infection. Conidial suspension (4×10^5^ conidia mL^-1^) of avirulent *M. oryzae* INA168 was inoculated onto the rice leaf sheaths in wild type (WT) rice HY and *ΔOsfer2* knock-out mutant lines. **(A)** Microscopic images of rice sheath epidermal cells infected with *M. oryzae.* hpi, hours post-inoculation. Scale bar=20 μm. **(B)** Quantification of invasive hyphae (IH) and hypersensitive response cell death (HR) in rice sheath cells infected with *M. oryzae* (48 hpi). Numbers of IH and HR cells were counted in rice sheath cells infected with *M. oryzae* (48 hpi). The values are means ± SD (n=4 leaf sheaths from different plants). Different letters above the bars indicate significantly different means (P<0.05), as analyzed by one-way ANOVA analysis. **(C)** Disease phenotypes on rice leaves infected with *M. oryzae* INA168. Three-week-old rice plants were sprayed with a conidial suspension (4×10^5^ conidia mL^-1^). Diseased leaves were photographed at 5 days after spray inoculation. hpi, hour post-inoculation; IH, invasive hyphae; HR, hypersensitive response.

We investigated whether *OsFER2* is required for cell death and resistant responses to *M. oryzae* INA168 infection using *ΔOsfer2* #3 and #4 plants (**Figure 5**). Avirulent *M. oryzae* INA168 grew poorly and caused HR cell death responses in leaf sheath epidermal cells of wild-type rice HY plants at 48 hpi (**Figures 5A**). However, *M. oryzae* INA168 grew well with invasive hyphae (IH) in the leaf sheath cells of *ΔOsfer2* #3 and #4. Avirulent *M. oryzae* INA168 infection induced significantly more HR cells in rice HY leaf sheaths than *ΔOsfer2* #3 and #4 leaf sheaths (**Figure 5B**). *M. oryzae* conidial suspension was spray-inoculated onto the leaves of three-week-old rice plants. Whole-leaf disease phenotypes were evaluated at 5 days after inoculation (**Figure 5C**). Rice HY leaves displayed no disease reaction or a typical resistant lesion with small pin-point necrotic spots during avirulent *M. oryzae* infection. By contrast, typical blast disease lesions appeared and enlarged in different greyish or whitish elliptical spots with brown or reddish-brown margins on the *ΔOsfer2* leaves. These results collectively indicate that *OsFER2* knock-out in rice HY induced susceptibility (disease) in response to avirulent *M. oryzae* infection. Moreover, there was no difference in disease phenotypes between *ΔOsfer2 #3 and #4* mutant plants. Thus, *ΔOsfer2 #3* was selected for use in further experiments.

### Time-course expression of defense-related genes in *ΔOsfer2* mutants during *M. oryzae* infection

**Figure 6:**
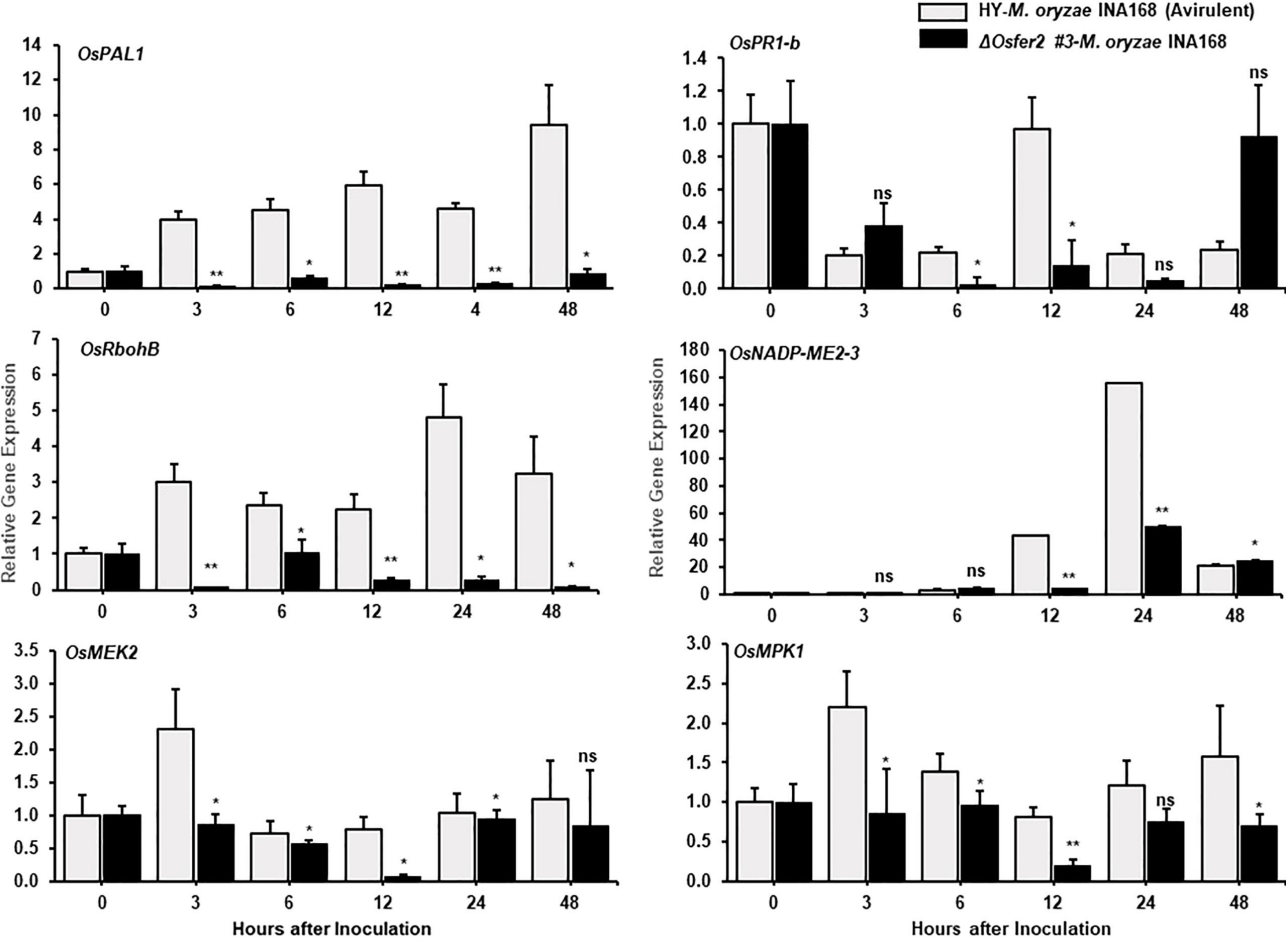
Quantitative real-time RT-PCR analyses of time-course expression of defense-related genes in rice HY and Δ*Osfer2* mutant plants during avirulent *Magnaporthe oryzae* INA168 infection. Expression of rice defense-related genes, such as respiratory burst oxidase homologue B (*OsRbohB*), phenylalanine ammonia-lyase-like, (*OsPAL1*), pathogenesis-related protein 1b (*OsPR1-b*), MAP kinase kinase 2 (*OsMEK2*), mitogen-activated protein kinase 1 (*OsMPK1)* and NADP-malic enzyme (*OsNADP-ME2-3*) were analyzed by qRT-PCR. Relative expression levels in rice leaf sheaths were calculated using the corresponding values at 0 hpi (control) after normalizing with respect to the expression of the internal control *OsUbiquitin.* Asterisks indicate statistically significant differences (Student’s *t*-test, *P< 0.05 and **P< 0.01), ns, not significant.

**Figure 7:**
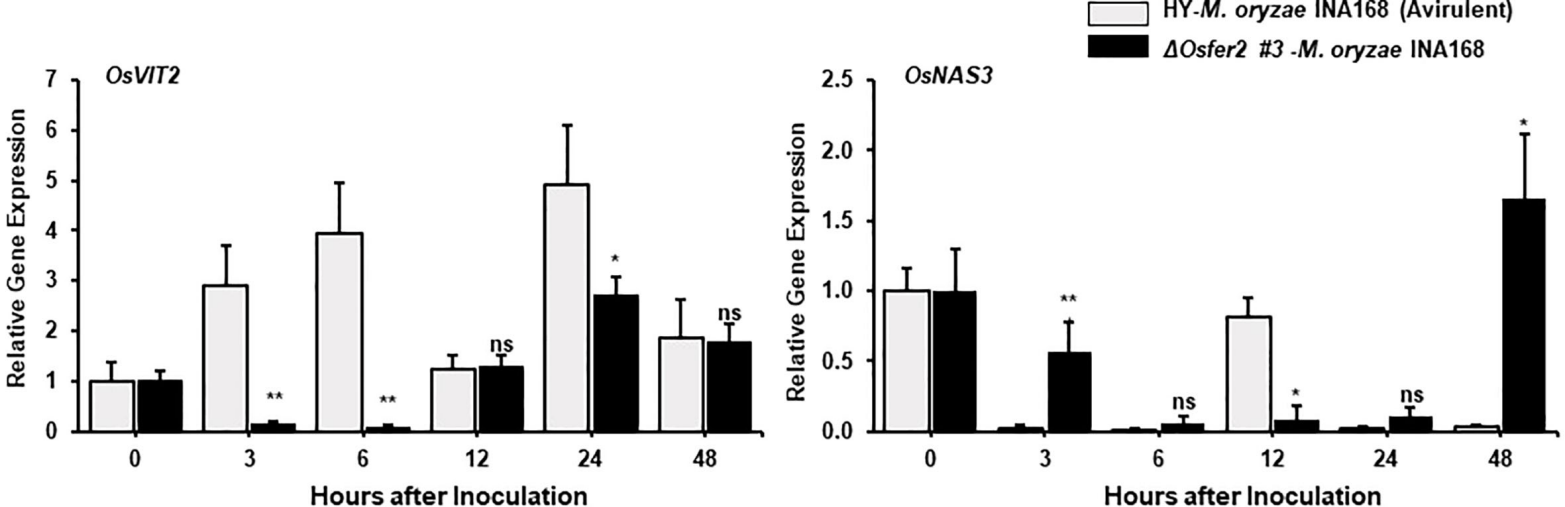
Quantitative real-time RT-PCR analysis of time-course expression of iron regulation genes in rice HY and Δ*Osfer2* mutant plants during avirulent *Magnaporthe oryzae* INA168 infection. Expression of rice iron regulation genes such as vacuolar Fe transporter 2 (*OsVIT2*) and NA synthase 3 (*OsNAS3*) were analyzed by qRT-PCR. Relative expression levels in rice leaf sheaths were calculated using the corresponding values at 0 hpi (control) after normalizing with respect to the expression of the internal control *OsUbiquitin.* Asterisks indicate statistically significant differences (Student’s *t*-test, *P<0.05 and **P<0.01). ns, not significant.

Some defense-related genes such as pathogenesis-related protein 1b (*OsPR1-b*), ascorbate peroxidase (*OsAPX1*), phenylalanine ammonia-lyase-like (*OsPAL1*) and probenazole-induced protein1 (*OsPBZ1*) have been demonstrated to play important roles in rice disease and immunity (Agrawal et al., 2003; Kim and Hwang, 2014; Wang et al., 2014; Kadota et al., 2015; Meng et al., 2019). In this study, we analyzed time-course expression levels of some defense-related genes, such as respiratory burst oxidase homologue B (*OsRbohB*), phenylalanine ammonia-lyase-like *(OsPAL1*), pathogenesis-related protein 1b (*OsPR1-b*), MAP kinase kinase 2 (*OsMEK2*), mitogen-activated protein kinase 1 (*OsMPK1)* and NADP-malic enzyme (*OsNADP-ME2-3*) in the leaf sheath tissues of rice HY and *ΔOsfer2* mutants during avirulent *M. oryzae* INA168 infection (**Figure 6**). Invariant expression of the internal control gene *OsUbiquitin* normalized expression levels of these defense-related genes in leaf sheaths of rice HY and *ΔOsfer2* mutants. Phenylalanine ammonia-lyase (PAL), an inducible enzyme, is involved in salicylic acid (SA)-dependent signaling of cell death and defense responses of plants to microbial pathogens (MacDonald and D’Cunha, 2007; Kim and Hwang, 2014). Avirulent *M. oryzae* INA168 infection distinctly induced *OsPAL1* expression in rice HY leaf sheaths (**Figure 6**). However, *OsPAL1* induction was significantly suppressed in *ΔOsfer2* leaf sheaths at all infection times (3-48 hpi), indicating that *OsFER2* knock-out suppressed *OsPAL1* induction in rice during infection. By contrast, avirulent *M. oryzae* INA168 infection did not significantly induce *OsPR1-b* expression in rice HY and *ΔOsfer2* leaf sheaths at the early infection time up to 48 hpi, except for lower expression at 6-12 hpi in *ΔOsfer2* leaf sheath (**Figure 7**). There also were no significant differences between rice HY and *ΔOsfer2* in expression of *OsAPX1* and *OsPBZ1* during *M. oryzae* infection (**Supplementary Figure 6**). *OsPBZ1* was known as a PBZ-inducible gene in rice (Nakashita et al., 2001).

In previous studies, we reported that rice respiratory burst oxidase homologue B (OsRbohB) and NADP-malic enzyme (OsNADP-ME) are involved in iron-and reactive oxygen species (ROS)-dependent ferroptotic cell death in rice-*M. oryzae* interactions (Dangol et al., 2019). Plant Rbohs that produce ROS are involved in plant disease and immunity (Morales et al., 2016; Jwa and Hwang, 2017; Dangol et al., 2021). The NADPH oxidase *OsRbohB* was distinctly downregulated at 3–48 hpi in *ΔOsfer2* leaf sheath, compared with high levels of *OsRbohB* induction in rice HY leaf sheaths (**Figure 6**). Similarly, avirulent *M. oryzae* INA168 infection did not induce *OsNADP-ME2-3* expression in *ΔOsfer2* leaf sheath at 12-24 hpi (**Figure 6**). These results indicate that *OsFER2* expression positively regulates *OsRbohB* and *OsNADP-ME* induction during avirulent *M. oryzae* infection.

Mitogen-activated protein kinase (OsMEK2 and OsMPK1) signaling is required for iron- and ROS-dependent ferroptotic cell death in rice (Dangol et al., 2021). Avirulent *M. oryzae* INA168 infection distinctly induced *OsMEK2* and *OsMPK1* expression at very early time (3 hpi) in rice HY leaf sheath (**Figure 6**). *OsFER2* knock-out significantly inhibited *OsMEK2* induction at 3-12 hpi in *ΔOsfer2* leaf sheath, compared to those in rice HY (**Figure 4**). Moreover, *OsMPK1* expression was lower in *ΔOsfer2* leaf sheath, at 3-48 hpi than those in rice HY (**Figure 6**). These data indicate that *OsFER2* expression is in part involved in mitogen-activated protein kinase (OsMEK2 and OsMPK1) signaling.

### Time-course expression of *OsVIT2* and *OsNAS3* in *ΔOsfer2* mutants during *M. oryzae* infection

The vacuolar membrane transporter 2 (OsVIT2) has been demonstrated to play an important role in sequestering Fe into the vacuole, and the *OsVIT2* gene was highly expressed in rice tissues under Fe excess conditions (Zhang et al., 2012; Finatto et al., 2015; Aung et al., 2018). Nicotianamine (NA) is a ubiquitous chelator of metal cations such as Fe^2+^ and Zn^2+^, and is responsible for metal homeostasis (Aung et al., 2019). Rice nicotianamine synthase (NAS) gene, *OsNAS3*, was strongly induced with excess Fe in most rice tissues, particularly rice old leaves (Aung et al., 2019). In our study, avirulent *M. oryzae* INA168 infection distinctly induced *OsVIT2* expression at very early time (3-24 hpi) in rice HY leaf sheath (**Figure 7**). However, *OsFER2* disruption strongly inhibited *OsVIT2* induction in *ΔOsfer2* rice sheaths at the early infection stage up to 24 hpi. In contrast, avirulent *M. oryzae* INA168 infection did not significantly induce *OsNAS3* expression in both HY and *ΔOsfer2* rice. These combined data indicate that *OsFER2* expression triggers *OsVIT2*, but not *OsNAS3* induction during avirulent *M. oryzae* infection.

### OsFER2 is required for ROS and ferric ion accumulation and lipid peroxidation during avirulent *M. oryzae* infection

**Figure 8:**
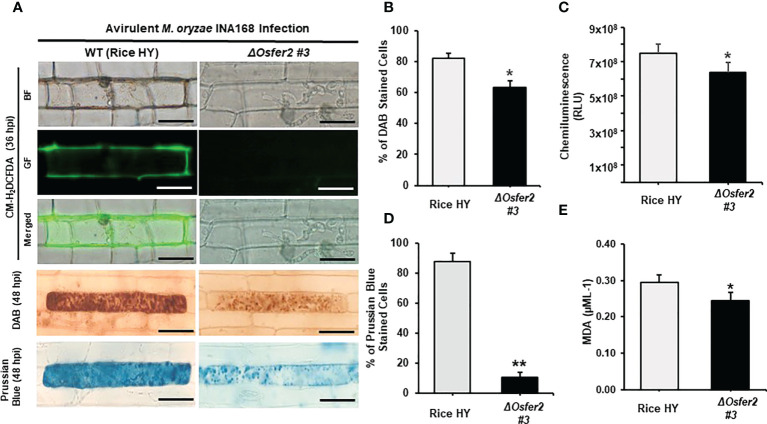
*OsFER2* knock-out in rice HY inhibits ROS and ferric ion (Fe^3+^) accumulation and lipid peroxidation in rice leaf sheaths infected with avirulent *Magnaporthe oryzae* INA168. **(A)** Microscopic images of epidermal cells of rice leaf sheaths stained with CM-H_2_DCFDA (green fluorescence), DAB, and Prussian Blue (Fe^3+^, blue color). Images were captured using a fluorescence microscope (Zeiss equipped with Axioplan 2) with bright field and a combination of excitation (450‒490 nm) and emission (515‒565 nm) GF filters. Scale bar=20 µm. **(B)** Quantification of DAB-stained cells, **(C)** ROS production, **(D)** Prussian Blue-stained cells, and **(E)** lipid (MDA) peroxidation at 48 hpi. Experiments were repeated three times with similar results. The values are means ± SD; *n*=4 leaf sheaths from different plants. Asterisks indicate statistically significant differences (Student’s *t-*test, *P<0.05 and **P<0.01). hpi, hour post-inoculation; HR, hypersensitive response; IH, invasive hypahae; BF, bright field; GF, green fluorescence; RLU, relative luminescent units; MDA, malondialdehyde.

In the recent study, we first reported that ferric ions and ROS are key factors triggering ferroptotic cell death in rice (Dangol et al., 2019). In this study, we further investigated whether rice *OsFER2* is involved in iron- and ROS-dependent ferroptotic cell death responses to avirulent *M. oryzae* infection in rice leaf sheaths using cytochemical staining techniques. CM-H_2_DCFDA and DAB staining was used to detect ROS in cells (Thordal‐Christensen et al., 1997; Kristiansen et al., 2009). By CM-H_2_DCFDA staining, green fluorescence, which reflects ROS accumulation in living cells, was detected in cell membrane and around invasive hyphae (IH) in wild-type rice HY, but not in *ΔOsfer2 #3* cells at 36 hpi (**Figure 8A**). In DAB staining, the dark brown products of the ROS and DAB reaction were clearly visible in cells of rice HY, whereas they were more brightly stained in *ΔOsfer2* #3 cells at 48 hpi (**Figure 8A**). The proportion of cells stained with DAB was significantly higher in rice HY sheaths, compared to *ΔOsfer2 #3* rice (**Figure 8B**). Similarly, the chemiluminescence assay using a luminometer revealed that ROS levels were higher in in rice HY sheaths, compared to *ΔOsfer2 #3* rice (**Figure 8C**).

In Prussian blue staining, the bright blue pigments (ferric ferrocyanides, which combine with Fe^3+^ inside leaf sheath epidermal cells) were clearly displayed in rice HY cells, but not in *ΔOsfer2 #3* rice cells at 48 hpi (**Figure 8A**). The proportion of Prussian blue-stained cells was significantly reduced in rice sheaths of *ΔOsfer2 #3*, compared to rice HY cells (**Figure 8D**). Lipid peroxidation levels in the leaf sheaths of rice HY and *ΔOsfer2 #3* at 48 hpi were also quantified using malondialdehyde (MDA) assay (**Figure 8E**). Lipid (MDA) peroxidation levels were significantly lower in *ΔOsfer2* rice sheaths than in rice HY. These results collectively indicate that *OsFer2* is involved in iron- and ROS-dependent ferroptotic cell death during avilrunt *M. oryzae* infection.

### The iron chelator DFO and ferroptosis inhibitor Fer-1 suppress ROS and Fe^3+^ accumulation and HR cell death in rice HY and *ΔOsfer2* mutant

The small-molecule iron chelator deferoxamine (DFO) and ferroptosis inhibitor ferrostatin-1 (Fer-1) have been demonstrated to inhibit iron- and ROS-dependent ferroptotic cell death in mammals (Dixon et al., 2012; Stockwell et al., 2017). DFO (3 mM) was treated onto the rice sheath epidermal layers at 42 h after inoculation with a conidial suspension of *M. oryzae* INA168 (4×10^5^ conidia mL^-1^). Infected rice leaf sheaths at 24 hpi was also treated and incubated with 10 µM Fer-1 solution.

**Figure 9:**
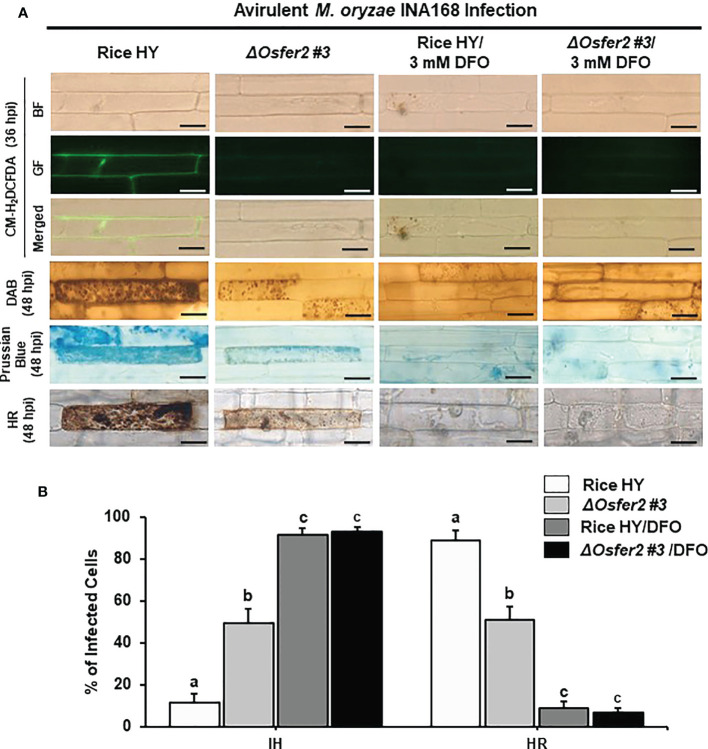
Treatment with deferoxamine (DFO) inhibits ROS and ferroptotic HR cell death in leaf sheath epidermal layers of rice HY and *ΔOsfer2* knock-out mutants infected with avirulent *Magnaporthe oryzae* INA168. Deferoxamine (DFO, 3 mM) was treated onto the epidermal layers at 42 h after inoculation with a conidial suspension (4×10^5^ conidia mL^-1^) of *M. oryzae* INA168. **(A)** Microscopic images of HR cell death, and DAB, CM-H_2_DCFDA and Prussian blue staining of rice sheath epidermal cells infected with *M. oryzae* INA168 and treated with DFO. The images are representative of different leaf sheath samples from three independent experiments. Scale bar = 20 µm. **(B)** Quantification of invasive hyphae (IH) and hypersensitive response cell death (HR) in rice sheath cells infected with *M. oryzae* INA168 and treated with DFO. Numbers of cells containing HR cell death and invasive hyphae were counted among rice sheath cells infected with *M. oryzae* at 48 hpi. The values are means ± SD; n=4 leaf sheaths from different plants. Different letters above the bars indicate significantly different means (*P*<0.05), as analyzed by one-way ANOVA analysis. hpi, hour post-inoculation; IH, invasive hypahae; HR, hypersensitive response; BF, bright field; GF, green fluorescence.

**Figure 10:**
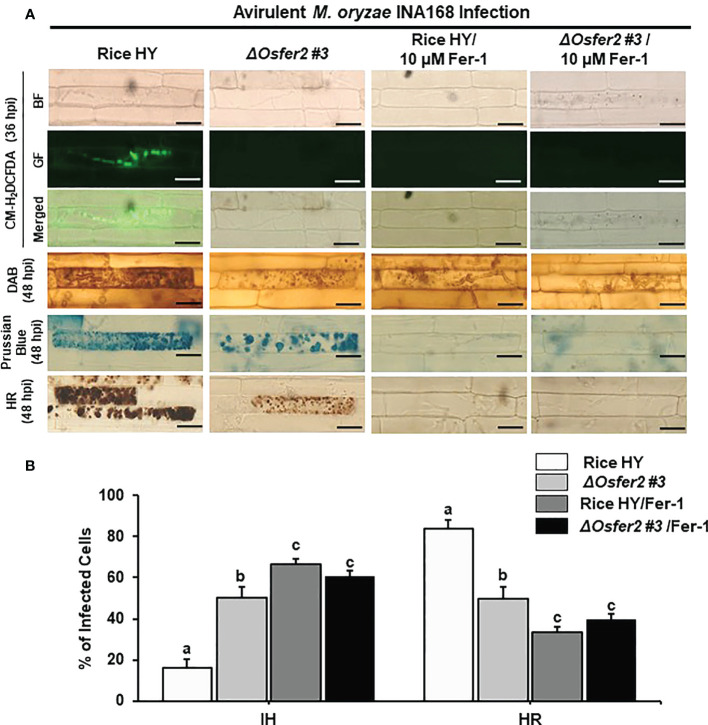
Treatment with ferrostatin-1 (Fer-1) inhibits ROS and ferroptotic HR cell death in the rice sheath epidermal layers of rice HY and *ΔOsfer2* knock-out mutants infected with avirulent *Magnaporthe oryzae* INA168. **(A)** Microscopic images of HR cell death, DAB, Prussian blue and CM-H_2_DCFDA staining of rice sheath epidermal cells infected with *M. oryzae* INA168 and treated with 10 µM Fer-1. Scale bar = 20 µm. **(B)** Quantification of invasive hyphae (IH) and hypersensitive response cell death (HR) in rice sheath cells infected with *M. oryzae* INA168 and treated with Fer-1. The rice sheath epidermal layers were incubated in 10 µM Fer-1 in dark condition at 25°C for 24 h after inoculation with *M. oryzae* conidia (4×10^5^ conidia mL^−1^). The values are means ± SD; n=4 leaf sheaths from different plants. Different letters above the bars indicate significantly different means (*P*<0.05), as analyzed by one-way ANOVA analysis. hpi, hour post-inoculation; HR, hypersensitive response; BF, bright field; GF, green fluorescence.

Treatment with DFO or Fer-1 inhibited the induction of ROS and Fe^3+^ accumulation and HR cell death in wild-type rice HY and *ΔOsfer2 #3* cells by avirulent *M. oryzae* INA168 infection, leading to successful colonization of IH in rice sheath cells (**Figures 9A**, **10A**). DFO and Fer-1 triggered the formation of normal hyphal structures of avirulent *M. oryzae* INA168 inside rice sheath cells. We further quantified the infected cell phenotypes (IH and HR) in the rice sheath epidermal layers treated with DFO or Fer-1 at 48 h after inoculation with avirulent *M. oryzae* INA168 (**Figures 9B**, **10B**). Rice leaf sheaths treated with DFO or Fer 1 had more cells with IH but fewer HR cells than the mock (water)-treated leaf sheaths in rice HY and *ΔOsfer2* #3 mutant during avirulent *M. oryzae* infection. These combined data indicate that DFO and Fer 1 inhibited the iron- and ROS-dependent accumulation to restrict HR cell death in both rice HY and *ΔOsfer2* knock-out mutants during avirulent *M. oryzae* INA168 infection.

### The actin microfilament inhibitor Cyt E and the redox inhibitor DPI suppress ROS and Fe^3+^ accumulation and HR cell death in rice HY and *ΔOsfer2* mutant

**Figure 11:**
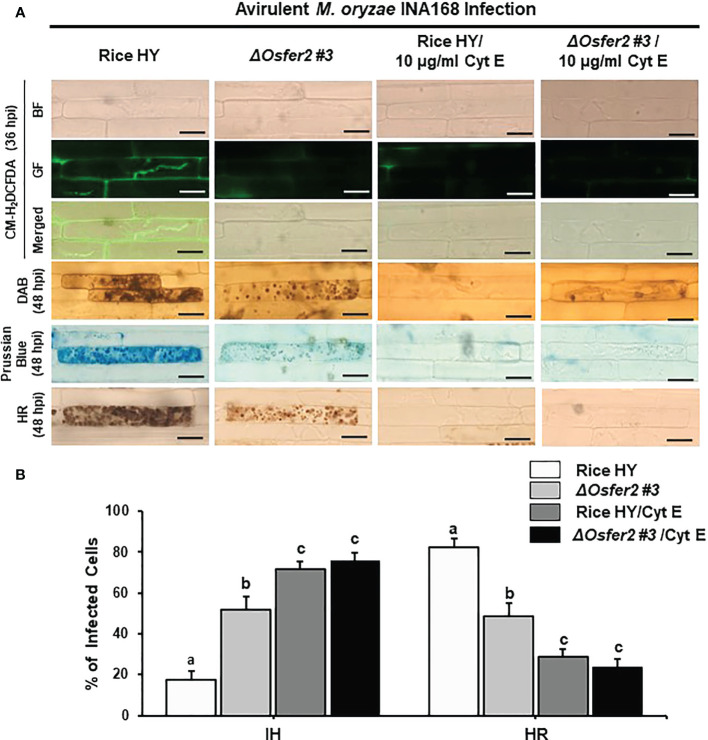
Treatment with cytochalasin E (Cyt E) inhibits ROS and ferroptotic HR cell death in the sheath epidermal layers of rice HY and *ΔOsfer2* knock-out mutants infected with avirulent *Magnaporthe oryzae* INA168. **(A)** Microscopic images of HR cell death, DAB, Prussian blue and CM-H_2_DCFDA staining of rice sheath epidermal cells infected with *M. oryzae* INA168 and treated with Cyt E (10 µg/ml). Scale bar=20 µm. **(B)** Quantification of invasive hyphae (IH) and hypersensitive response (HR) cell death in rice sheath cells inoculated with *M. oryzae* and treated with Cyt E The rice sheath epidermal layers were incubated in 10 µg/ml cytochalasin E (Cyt E) for 24 h in dark condition at 25°C after inoculation with *M. oryzae* conidia (4×10^5^ conidia mL^−1^). The values are means ± SD; n=4 leaf sheaths from different plants. Different letters above the bars indicate significantly different means (P<0.05), as analyzed by one-way ANOVA analysis. hpi, hour post-inoculation; HR, hypersensitive response; BF, bright field; GF, green fluorescence.

**Figure 12:**
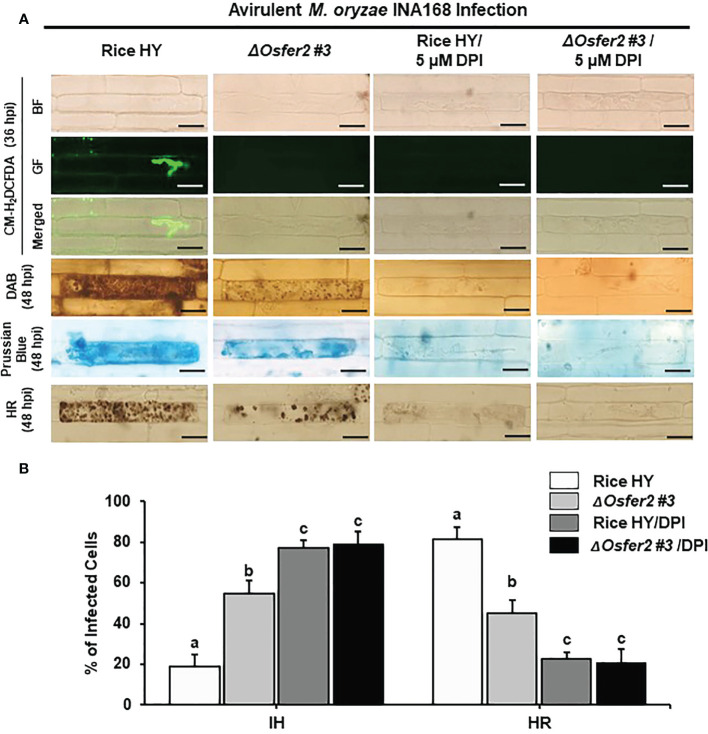
Treatment with the redox inhibitor diphenyleneiodonium (DPI) inhibits ROS and ferroptotic HR cell death in the rice sheath epidermal layers of rice HY and *ΔOsfer2* knock-out mutants infected with avirulent *Magnaporthe oryzae* INA168. **(A)** Microscopic images of HR cell death, DAB, Prussian blue and CM-H_2_DCFDA staining of rice sheath epidermal cells infected with *M. oryzae* INA168 and treated with DPI (5 µM). Scale bar = 20 µm. **(B)** Quantification of invasive hyphae (IH) and hypersensitive response cell death (HR) in rice sheath cells infected with *M. oryzae* INA168 and treated with DPI. DPI was treated with *M. oryzae* conidia (4×10^5^ conidia mL^−1^) to the rice leaf sheaths, followed by 48 h-incubation at 25°C in dark condition. The values are means ± SD; n=4 leaf sheaths from different plants. Different letters above the bars indicate significantly different means (P<0.05), as analyzed by one-way ANOVA analysis. hpi, hour post-inoculation; HR, hypersensitive response; BF, bright field; GF, green fluorescence.

Cyt E inhibits actin microfilament polymerization in plant cells (Yun et al., 2003; Shimada et al., 2006). The redox inhibitor DPI also inhibits the plasma membrane NADPH oxidase activity required to generate extracellular ROS in plant cells (Morré, 2002; Kadota et al., 2015). In this study, we investigated whether Cyt E and DPI regulate ROS (H_2_O_2_), Fe^3+^ accumulation and HR cell death in rice leaf sheaths of wild-type rice HY and *ΔOsfer2 #3* during avirulent *M. oryzae* INA168 infection. The rice sheath epidermal layers were incubated in Cyt E (10 µg/ml) for 24 h after inoculation with *M. oryzae* (4×10^5^ conidia mL^-1^). The redox inhibitor DPI (5 µM) was also treated together with *M. oryzae* conidial suspension to the rice leaf sheaths. Rice sheath epidermal cells were stained with CM-H_2_DCFDA and DAB for ROS detection and Prussian blue for Fe^3+^ detection (**Figures 11A**, **12A**).

Avirulent *M. oryzae* INA 168 infection induced strong ROS and Fe^3+^ accumulation and HR cell death with dark brown cellular aggregates in the pathogen invaded cells of rice HY leaf sheaths (**Figures 11A**, **12A**). However, treatment with Cyt E and DPI distinctly inhibited ROS and Fe^3+^ accumulation and HR cell death in both rice HY and *ΔOsfer2 #3* mutant, leading to the successful colonization of IH inside rice leaf sheath cells during avirulent *M. oryzae* INA 168 infection (**Figures 11A**, **12A**). Rice leaf sheaths treated with Cyt E and DPI had more cells with IH but fewer HR death cells than did the mock (water)-treated leaf sheaths in both wild-type rice HY and *ΔOsfer2 #3* mutant at 48 h after inoculation with avirulent *M. oryzae* INA 168 (**Figures 11B**, **12B**). These combined data indicate that the actin microfilament inhibitor Cyt E and the redox inhibitor DPI suppressed the iron-and ROS-dependent accumulation and HR cell death in both rice HY and *ΔOsfer2* knock-out mutants during avirulent *M. oryzae* INA168 infection.

### The small-molecule inducer erastin does not trigger ROS and Fe^3+^ accumulation and HR cell death in *ΔOsfer2* mutants

**Figure 13:**
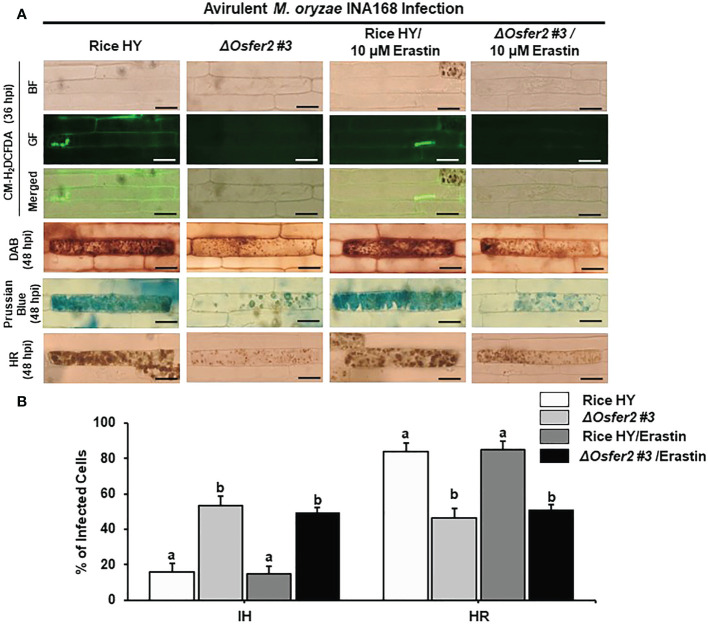
Treatment with erastin does not induce iron- and ROS-dependent ferroptotic cell death in rice sheath epidermal layers of *ΔOsfer2* knock-out mutants during avirulent *Magnaporthe oryzae* INA168 infection. **(A)** Images of HR cell death, DAB, Prussian blue and CM-H_2_DCFDA staining of rice sheath epidermal cells infected with *M. oryzae* INA168 and treated with erastin (10 µM). Scale bar=20 µm. **(B)** Quantification of invasive hyphae IH and hypersensitive response (HR) cell death in rice sheath cells infected with *M. oryzae* INA168 and treated with erastin. *M. oryzae* conidia (4×10^5^ conidia mL^−1^) were mixed with 10 mM erastin and then inoculated on leaf sheaths, followed by 48 h-incubaion at 25°C in dark condition. The values are means ± SD; n=4 leaf sheaths from different plants. Different letters above the bars indicate significantly different means (*P*<0.05), as analyzed by one-way ANOVA analysis. hpi, hour post-inoculation; HR, hypersensitive response; BF, bright field; GF, green fluorescence.

Erastin is a small-molecule inducer of iron-dependent ferroptotic cell death in mammalian cells (Dixon et al., 2012) and plant cells (Dangol et al., 2019; Dangol et al., 2021). In this study, we investigated whether erastin treatment regulates ROS, Fe^3+^ accumulation and HR cell death in rice leaf sheaths of *ΔOsfer2 #3* knock-out mutants during avirulent *M. oryzae* INA168 infection. Erastin (10 µM) was mixed with *M. oryzae* conidial suspension and treated onto rice sheaths. CM-H_2_DCFDA (GF), DAB and Prussian blue staining showed that erastin treatment did not enhance ROS and Fe^3+^ accumulation and HR cell death in the leaf sheath epidermal layers of rice HY and *ΔOsfer2* knock-out mutant infected with *M. oryzae* INA168 (**Figure 13A**). Furthermore, erastin treatment did not significantly reduce the number of cells with IH, but also did not increase the number of cells with HR, in leaf sheaths of rice HY and *ΔOsfer2* knock-out mutants infected with *M. oryzae* IHA168 (**Figure 13B**). These combined data indicate that the small-molecule inducer of ferroptosis erastin does not trigger iron- and ROS-dependent induction of ferroptotic cell dealth in *ΔOsfer2* mutant plants during avirulent *M. oryzae* infection.

### *OsFER2* complementation in *ΔOsfer2* mutants restores ROS and ferric Ion Accumulation and HR Cell Death Phenotypes

**Figure 14:**
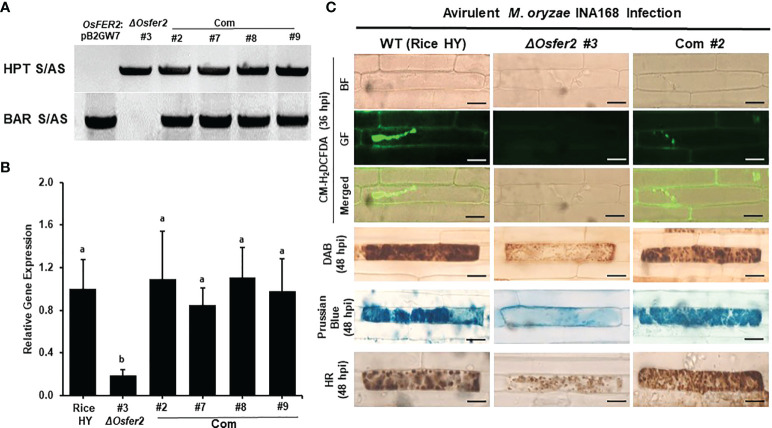
*OsFER2* complementation in *ΔOsfer2* #3 mutants restores ROS and ferric ion (Fe^3+^) accumulation and HR cell death phenotypes in rice leaf sheaths infected with avirulent *Magnaporthe oryzae* INA168. **(A)** Identification of *OsFER2* complementation in *ΔOsfer2 #3* mutants. Hygromycin primers (HPT S/AS) were used to identify hygromycin plant selection protein in *ΔOsfer2* background plants. BAR primers (BAR S/AS) was used to verify the presence of BAR plant selection protein in *OsFER2* complementation plants. The plasmid OsFER2:pB2GW7 and gDNA of *ΔOsfer2* were used as controls. **(B)** Relative *OsFER2* expression in rice HY, *ΔOsfer2* and *OsFER2* complementation plants. *OsFER2* expression was normalized using the expression of the internal control *OsUbiquitin.* Different letters above the bars indicate significantly different means (*P*<0.05), as analyzed by one-way ANOVA analysis. **(C)** Microscopic images of rice leaf sheath epidermal cells stained with CM-H_2_DCFDA (green fluorescence), DAB and Prussian blue (Fe^3+^, blue color) during avirulent *M. oryzae* INA168 infection. Images were captured using a fluorescence microscope (Zeiss equipped with Axioplan 2) with bright field and a combination of excitation (450‒490 nm) and emission (515‒565 nm) GF filters. The images shown are representative of the different leaf sheath samples that were observed in three independent experiments. hpi, hour post-inoculation; HR, hypersensitive response; BF, bright field; GF, green fluorescence. Scale bar=20 µm.

To validate that *OsFER2* is the causal gene for *ΔOsfer2* mutants, we created the *OsFER2* complementation plants through *Agrobacterium*-mediated transformation by transferring full-length *OsFER2* coding sequence into *ΔOsfer2* #3 mutant rice calli using the *OsFER2:*:pB2GW7 binary vector (**Supplementary Figure 6**). All the *ΔOsfer2*::pB2GW7, *ΔOsfer2 #3*, and *OsFER2* complementation lines were verified for the HPT selection gene in *ΔOsfer2* background and the BAR selection gene in the complementation vector pB2GW7 (**Figure 14A**). RT-PCR and real-time qRT-PCR assays showed that *OsFER2* was not expressed in the *ΔOsfer2 #3* mutant, but highly upregulated in the *OsFER2* complementation lines #2, #7, #8 and #9 during avirulent *M. oryzae* INA168 infection (**Figure 14B** **;** **Supplementary Figures 7A, B** **)**. Levels of *OsFER2* expression were normalized by invariant expression of the internal control genes *OsUbiquitin*, *18S rRNA* and *OsActin* (**Supplementary Figures 7A, B** **)**. These results indicate that *OsFER2* expression was restored in *OsFER2* complementation lines.

We further investigated ROS and ferric ion (Fe^3+^) accumulation and HR cell death in leaf sheath cells of rice HY, *ΔOsfer2* mutants and *OsFER2* complementation lines during avirulent *M. oryzae* INA168 infection (**Supplementary Figure 8** **;** **Figure 14C**). CM-H_2_DCFDA (green fluorescence), DAB (dark brown) and Prussian blue (Fe^3+^, blue color) staining revealed that ROS (H_2_O_2_) and ferric ion (Fe^3+^) strongly accumulated inside and around invasive hyphae (IH) leading to the HR cell death in leaf sheath cells of rice HY and *OsFER2* complementation lines at 36–48 hpi, compared to *ΔOsfer2 #3* mutants. These results indicate that *OsFER2* complementation restores the iron- and ROS-dependent ferroptotic cell death phenotype in *ΔOsfer2* mutants.

## Discussion

Ferroptosis, a form of non-apoptotic cell death, is dependent on intracellular iron, but not other metals (Dixon et al., 2012). Ferroptotic cell death occurs in rice as an immune response to block *Magnaporthe oryzae* infection (Dangol et al., 2019). In plants, ferritin is a ubiquitous iron storage protein that buffers iron inside cells (Harrison and Arosio, 1996; Briat and Lobréaux, 1997; Briat et al., 2010). It is well documented that rice ferritin protein acts as an iron buffer in the defense response of rice to iron-or metal-mediated oxidative stress (Silveira et al., 2009; Stein et al., 2009). Exposure to copper, methyl viorgen (Paraquat), sodium nitroprusside (SNP) and excess iron induced *OsFER1* and *OsFER2* expression in rice plants. In this study, we demonstrated that rice ferritin 2 (OsFER2) positively regulates iron- and ROS-dependent ferroptotic cell death in rice-*M. oryzae* interactions.

### Structures of rice ferritin and other plant ferritin proteins

In the present study, we cloned and sequenced rice ferritin genes *OsFER1* and *OsFER2*. Base changes in the *OsFER2* cDNA sequence were insignificant, based on the RGAP (Rice Genome Annotation Project); consequently, the changes in the nucleotide sequence did not affect the amino acid level or functional region of the *OsFER2* gene. In addition, the alternative splicing forms of *OsFER2* have been clarified. The nucleotide and deduced amino acid sequences of rice ferritin proteins provide fundamental information that underlies rice ferritin structure, subcellular localization and biochemical function. The sequence of the ferritin protein is highly conserved in plants. In the N-terminal region, plant ferritins contain plant-specific transit peptides responsible for targeting of precursor proteins to plastids (Ragland et al., 1990; Briat et al., 1999; Bruce, 2000). The second part of plant ferritins, called the mature region, contains an extension peptide, four helix bundles, and a short C-terminal helix. Extension peptides are important for plant ferritin protein stability (Van Wuytswinkel et al., 1995; Briat and Lobréaux, 1997; Briat et al., 2010; Masuda et al., 2012). The four helix bundles are located in functional sites that establish ferroxidase diiron center, ferrihydrite nucleation center and iron ion channel (Harrison and Arosio, 1996). *OsFER2* is known as a key gene of rice ferritin against iron-mediated oxidative stress (Stein et al., 2009).

### Localization of OsFER2 and its transit peptide domain to the chloroplast

In plants, chloroplast is the most Fe-rich organelle for photosynthesis and contains 80~90% of iron required for photosynthesis in leaves (Terry and Low, 1982; Vigani et al., 2013). In this study, we investigated subcellular localization of the full-length ferritin protein OsFER2 and its transit peptides and mature regions. Our results showed that the transit peptide itself and the full-length ferritin OsFER2 were localized to chloroplasts, whereas the mature regions of OsFER2 were ubiquitous localized in cells. This suggests that the transit peptide acts as a signal peptide for the ferritin protein to target chloroplasts. Rice ferritin transit peptide may act as a chloroplast-targeting sequence. The ferritin 2 protein is encoded by nuclear DNA and synthesized in the cytoplasm as a ferritin precursor (Van der Mark et al., 1983). The transit peptide drives the ferritin precursor to the chloroplast and delivers the ferritin 2 protein to the organelle, followed by cleavage inside the chloroplast (Bruce, 2000; Teixeira and Glaser, 2013; Kumar et al., 2018). In chloroplasts, mature region of the ferritin protein assembled with others to form the 24-subunit mature ferritin protein (Ragland et al., 1990; Briat et al., 2010; Yang et al., 2015). These results support the possibility that ferritin is a major Fe storage molecule in chloroplasts (Ravet et al., 2009; Kroh and Pilon, 2020). Taken together, we suggest that rice ferritin protein plays a crucial role in iron storage in chloroplasts.

Iron homeostasis is tightly maintained in plant cells by a number of closely linked exclusion and inclusion adaptation strategies (Gross et al., 2003; Quinet et al., 2012; Finatto et al., 2015; Aung and Masuda, 2020). In rice, vacuoles are another organelle for Fe storage in cells. The vacuolar membrane transporter OsVIT2 has been identified as a major channel protein that regulates iron trafficking between the cytoplasm and the vacuole (Ragland et al., 1990; Aung et al., 2018). Nicotianamine (NA) is a plant-derived chelator of various divalent cations in plants. Plants maintain metal homeostasis by its chelation, and utilize it for transport of metal cations, including iron (Hell and Stephan, 2003; Takahashi et al., 2003). Nicotianamine is biosynthesized by NA synthases (Higuchi et al., 1994; Zhang et al., 2012). Rice NA synthase, OsNAS3, is closely involved in the response to Fe excess (Aung et al., 2018; Aung et al., 2019). Expression of *OsFER*s, *OsVIT2* and *OsNAS3* was highly induced in excess Fe conditions (Aung et al., 2018). However, in our study, *OsFER2* knock-out suppressed *OsVIT2* but not *OsNAS3* expression in *ΔOsfer2* mutants during avirulent *M. oryzae* INA168 infection. These results suggest that *OsFER2* expression differentially regulates iron regulatory genes, such as *OsVIT2* and *OsNAS3* in rice cells.

### *OsFER2* expression induces iron and ROS accumulation and ferroptotic cell death

Plant cells require an optimal Fe concentration to successfully complete their life cycle (Guerinot and Yi, 1994), but it could not exceed certain thresholds that cause toxicity to the cell (Pierre and Fontecave, 1999). Thus, plants accept excess Fe into ferritin proteins to tightly control Fe homeostasis by accommodating excess Fe into ferritin proteins (Briat et al., 2006; Briat et al., 2010). In host-pathogen interactions, there is a battle between the host cell and the pathogen for the essential nutrient irons. The outcome of host-pathogen competition is the successful pathogen infection of the host or host resistance to pathogen (Nairz et al., 2010). The experimental evidence that plants used ferritin as a weapon to inhibit pathogen growth is well documented. Plant polyphenols inhibit the growth of *Erwinia chrysanthemi* in plants by mimicking iron-binding proteins in animals (Mila et al., 1996; Zhang et al., 2012). Expression of the ferritin genes was upregulated in potatoes during *Phytophthora infestans* infection (Mata et al., 2001) and in *Arabidopsis* during *Erwinia chrysanthemi* infection (Dellagi et al., 2005). In line with this, ferritin protects genetically modified tobacco cells from oxidative damage and pathogen attack (Deák et al., 1999). Overall, the role of ferritin in immunity is further emphasized.

In the incompatible interaction between rice and *M. oryzae*, iron ions and ROS are highly accumulated in rice leaf sheath tissue to cause iron- and ROS-dependent ferroptotic cell death (Dangol et al., 2019). In our present study, we found that avirulent *M. oryzae* infection distinctly induced *OsFER2* expression in rice HY leaf sheaths. *OsFER2* knock-out induced less HR cell death, and more severe blast disease in *ΔOsfer2* mutant plants. This suggests that OsFER2 has a cell death-regulatory function in rice. ROS is known to be an essential factor for cell death against pathogen infection (Apel and Hirt, 2004). *OsFER2* expression induced ROS and ion accumulation and lipid peroxidation in rice cells during avirulent *M. oryzae* infection. However, *OsFER2* knock-out inhibited ROS accumulation in rice cells. This resulted in reduced levels of lipid peroxidation (Zoeller et al., 2012). In this context, the intracellular iron intensity detected by Prussian blue staining in infected cells was faded in *ΔOsfer2* leaf sheath cells, compared to wild type rice HY. However, *OsFER2* complementation in the *ΔOsfer2* mutant induced strongly stained ROS and iron intensity in *M. oryzae*-infected cells, suggesting that *OsFER2* function was fully restored to wild-type levels. Taken together, these results suggest that *OsFER2* expression is involved in iron- and ROS-dependent ferroptotic cell death during avirulent *M. oryzae* infection.

### OsFER2 and rice defense-related genes in rice ferroptotic cell death

Rice respiratory burst oxidase homologue B (OsRbohB) and NADP-malic enzyme (OsNADP-ME) are required for the generation of ROS in plant cells (Mittler et al., 2004; Dangol et al., 2019). In our study, *OsRbohB* and *OsNADP-ME* was significantly downregulated in *ΔOsfer2* knock-out mutant plants. This suggests that rice ferritin protein positively regulates intraculular ROS production in rice-*M. oryzae* interaction *via* OsRbohB and OsNADP-ME. It has been well demonstrated that mitogen-activated protein (MAP) kinase (MAPK) signaling pathways play a crucial role in plant defense (Ishihama et al., 2011; Melech-Bonfil and Sessa, 2011; Meng and Zhang, 2013; Oh et al., 2013; Thulasi Devendrakumar et al., 2018). *OsFER2* deficiency in *ΔOsfer2* knock-out plants resulted in decreased expression of *OsMPK1* and *OsMEK2*, suggesting that rice ferritin *OsFER2* modulates the *OsMPK1* and *OsMEK2* signaling pathways in rice during *M. oryzae* infection. Our recent studies demonstrated that *OsMPK1* and *OsMEK2* play an important role in iron- and ROS-dependent ferroptotic cell death in rice (Dangol et al., 2021). These combined data suggest that Fe stored in ferritin tightly regulates the expression of some defense-related genes such as *OsPAL1*, *OsRbohB*, *OsNADP-ME2-3*, *OsMEK2* and *OsMPK1*. However, further studies are required to determine why there are no significant differences between rice HY and *ΔOsfer2* in the expression of *OsPR1-b, OsAPX1* and *OsPBZ1* during *M. oryzae* infection.

In previous studies, the actin microfilament polymerization inhibitor Cyt E (Yun et al., 2003; Shimada et al., 2006) suppressed Fe^3+^ and ROS accumulation inside and around IH of avirulent *M. oryzae* (Dangol et al., 2019). In this study, inhibition of ferroptotic cell death by Cyt E supports the possibility that plants have developed different cellular mechanisms to maintain iron homeostasis in addition to the uptake of iron into ferritin (Gross et al., 2003; Quinet et al., 2012; Finatto et al., 2015; Aung and Masuda, 2020). In this context, treatment with the iron chelator desferrioxamine (DFO) significantly reduced the number of cell death in *ΔOsfer2* plants to a level similar to that of rice HY. In mice, DFO inhibits NADPH oxidase-dependent ROS production by chelating active site heme iron, blocking electron transfer from NADPH to oxygen and its reduction to O^2-^ (Dovhanj et al., 2010). ROS produced by NADPH oxidases (Rbohs) may be required for ROS-dependent ferroptotic cell death (Dixon et al., 2012; Singh et al., 2016). *OsFER2* knock-out in *ΔOsfer2* affected *OsRhohB* and *OsNADP-ME* expression. Treatment with the NADPH oxidase inhibitor diphenyleneiodonium (DPI) significantly inhibited HR cell death. These results indicate that *OsFER2* knock-out is accompanied by a decrease in ROS, leading to the inhibition of ferroptotic cell death. The lipid ROS scavenger Fer-1 treatment reduced cell death by 10% in *ΔOsfer2* plants. All major pathways generating lipid ROS require iron (Lei et al., 2019). Accordingly, lipid ROS was important for ferroptotic cell death (Dixon et al., 2012; Dangol et al., 2019; Dangol et al., 2021). These combined results indicate that DFO, Fer 1, Cyt E and DPI suppress the iron-and ROS-dependent accumulation to restrict HR cell death in both rice HY and *ΔOsfer2* mutants during avirulent *M. oryzae* infection. The data also support the possibility that Fe stored in ferritin is not a unique source of intracellular iron and contributes significantly to various cellular metabolism, leading to ferroptotic cell death. The small molecule inducer erastin enhanced Fe^3+^ and ROS accumulation and inhibited cellular glutathione production, resulting in ferroptotic cell death independent of NADP-ME2 (Dixon et al., 2012; Dangol et al., 2019). In this study, however, erastin did not enhance ferroptotic cell death in *ΔOsfer2* plants. That is because *OsFer2* knock-out significantly reduces the intracellular iron source required for ferroptosis, so even if more ROS is generated by erastin, ferroptosis could not be boosted.

### Proposed model of OsFER2 signaling for ferroptotic cell death in rice-*M. oryzae* interactions

**Figure 15:**
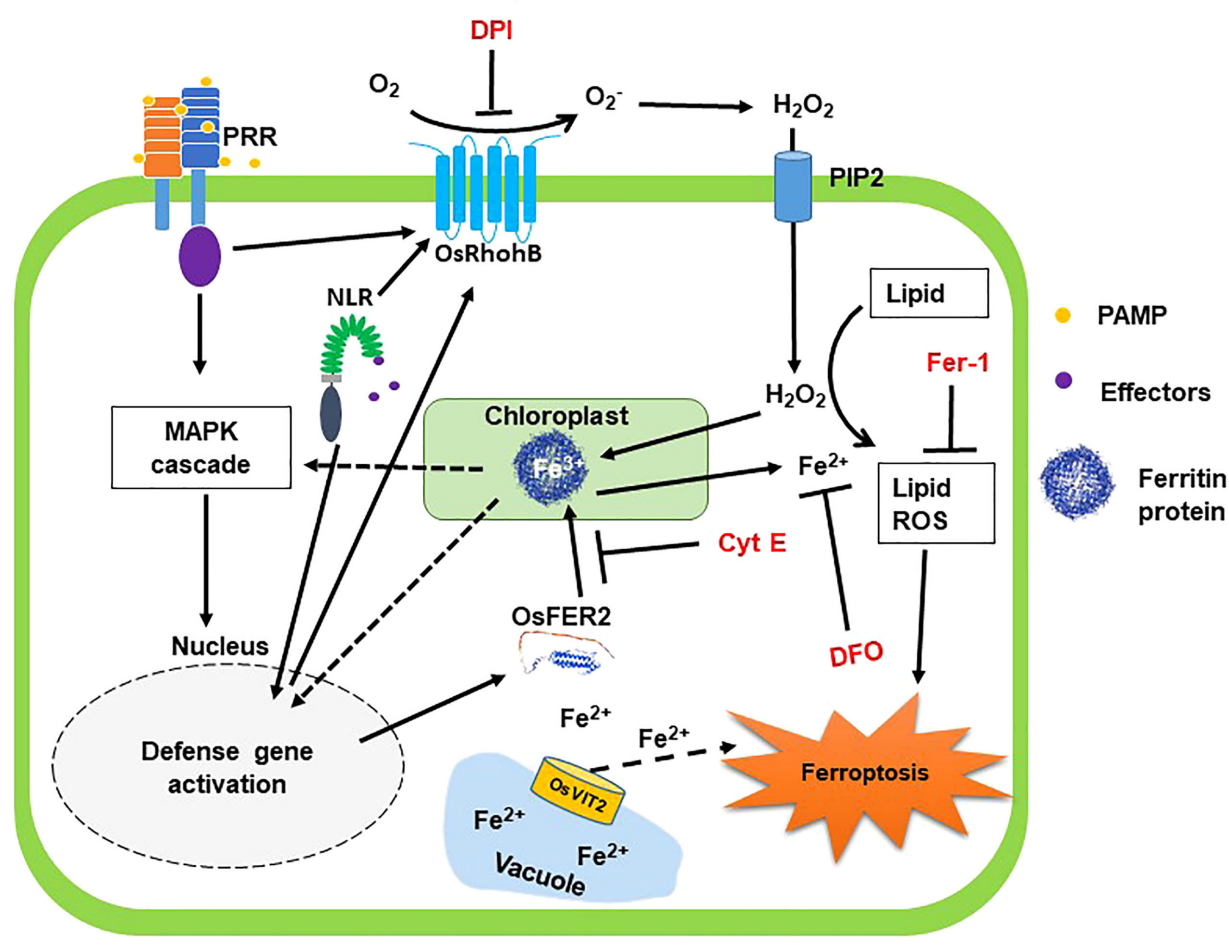
Proposed model of rice iron-storage protein ferritin 2 (OsFER2) signaling for iron- and ROS-dependent ferroptotic cell death in rice–*Magnaporthe oryzae* interactions. During avirulent *M. oryzae* infection, perception of PAMPs or pathogen effectors *via* membrane-bound PRRs or NLRs, respectively, triggers OsMAP kinase signaling to the nucleus to induce defense genes in rice cells. Iron is stored in vacuoles and ferritins. OsFER2 is localized to the chloroplast. OsFER2 positively regulates the vacuolar membrane transporter 2, OsVIT2, to maintain iron homeostasis in cells. Iron stored in ferritin contributes to lipid-ROS formation leading to ferroptotic cell death. DFO acts as an iron chelator, adsorbing iron inside the cell. Cyt E inhibits actin microfilament polymerization in plant cells. Fer-1 blocks lipid peroxidation and DPI inhibits NADPH oxidase activity. The small-molecule ferroptosis inhibitors DFO, Cyt E, Fer-1 and DPI are in red. Solid arrows and solid T-shaped lines indicate positive and negative regulations, respectively. Dotted arrows indicate indirect or unverified connections. NLR, nucleotide-binding leucine-rich repeat; PAMP, pathogen-associated molecular pattern; PRR, pattern recognition receptor; PIP2, aquaporin channel.

Based on the results obtained in this study, we propose a working model of rice iron-storage protein ferritin 2 (OsFER2) signaling to regulate iron- and ROS-dependent ferroptotic HR cell death in rice–*M. oryzae* interactions (**Figure 15**). Avirulent *M. oryzae* INA168 infection significantly induced *OsFER2* expression and HR resistant response in rice leaves. Recognition of PAMPs or *M. oryzae* effectors *via* membrane-bound PRRs or NLRs (Jones and Dangl, 2006; Zipfel, 2008) may activate OsMAP kinase cascade in rice cells to activate defense-related genes such as *OsFER2* in rice cells. OsFER2, the major subunit of rice ferritin, stores and releases iron atoms in the native ferritin cage (Briat et al., 2010; Yang et al., 2015). OsFER2 is localized to the chloroplast. Iron is stored in vacuoles and ferritins. Ferritin acts as an iron storage protein, maintaining intracellular iron homeostasis (Briat et al., 2010). Iron released from OsFER2 may be required for activation of MAP kinase signaling (OsMEK2, OsMPK1), signaling upstream of OsRbohB, and expression of defense-related genes (*OsPAL1, OsPR1-b*) (Dangol et al., 2021). Ferritin’s iron also contributes significantly to the formation of lipid ROS, a major factor in ferroptosis (Dixon et al., 2012; Dangol et al., 2019; Dangol et al., 2021). Iron loss from ferritin protein, which cause iron deficiency, may affect various defense mechanisms of cells, such as the activation of OsRbohB and OsNADP-ME and the generation of lipid ROS. The small-molecule ferroptosis inhibitors DFO, Cyt E and Fer-1 and the redox inhibitor DPI suppress the accumulation of intracellular iron and ROS, which prevent HR cell death response in rice cells. *OsFER2* knock-out and consequent reduction in ROS production in rice cells inhibit iron- and ROS-dependent ferroptotic HR cell death and the immune response to *M. oryzae* infection. In conclusion, *OsFER2* expression positively regulates ferroptotic cell death in the rice-*M. oryzae* interaction.

## Data availability statement

The original contributions presented in the study are included in the article/**Supplementary Material**. Further inquiries can be directed to the corresponding author.

## Author contributions

Study conception and design, N-SJ; data collection, NN, JW, and DL; analysis and interpretation of results, NN; writing and original draft preparation, NN, BH, and N-SJ; review and editing, BH and N-SJ. All authors contributed to the article and approved the submitted version.

## Funding

This work was performed with financial support from the Cooperative Research Program for Agriculture Science and Technology Development (Project No. PJ015966012021), Rural Development Administration, Republic of Korea and the National Research Foundation (NRF) of Korea (Grant No. 2022R1F1A1074318).

## Conflict of interest

The authors declare that the research was conducted in the absence of any commercial or financial relationships that could be construed as a potential conflict of interest.

## Publisher’s Note

All claims expressed in this article are solely those of the authors and do not necessarily represent those of their affiliated organizations, or those of the publisher, the editors and the reviewers. Any product that may be evaluated in this article, or claim that may be made by its manufacturer, is not guaranteed or endorsed by the publisher.
