# Supplementary material for: Rice iron storage protein ferritin 2 (OsFER2) positively regulates ferroptotic cell death and defense responses against Magnaporthe oryzae
Source: Front Plant Sci. 2022 Oct 24;13:1019669. doi: 10.3389/fpls.2022.1019669 (PMC9639352; doi:10.3389/fpls.2022.1019669)
Supplement: Supplementary file 2 [file DataSheet_1.pdf]

A

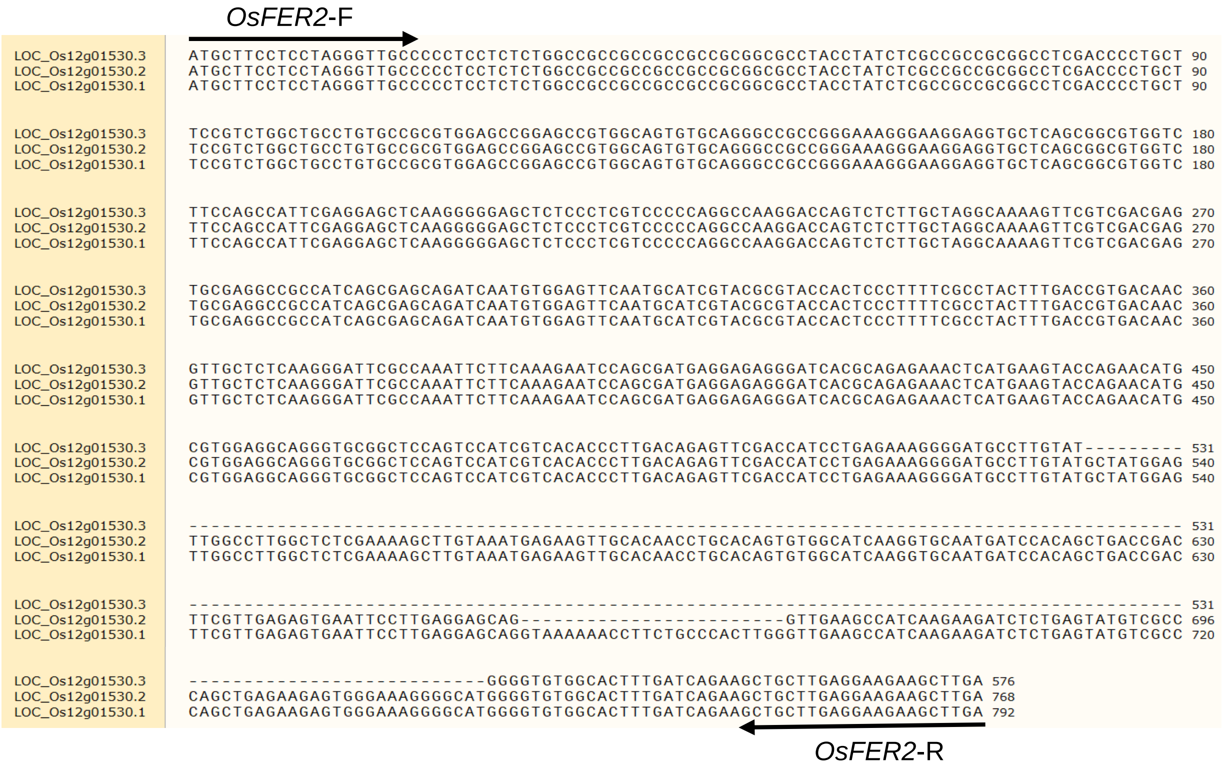

B

	LOC_Os12g01530.3	LOC_Os12g01530.2	LOC_Os12g01530.1
**LOC_Os12g01530.3**	100.00	96.53	96.53
**LOC_Os12g01530.2**	96.53	100.00	100.00
**LOC_Os12g01530.1**	96.53	100.00	100.00

C

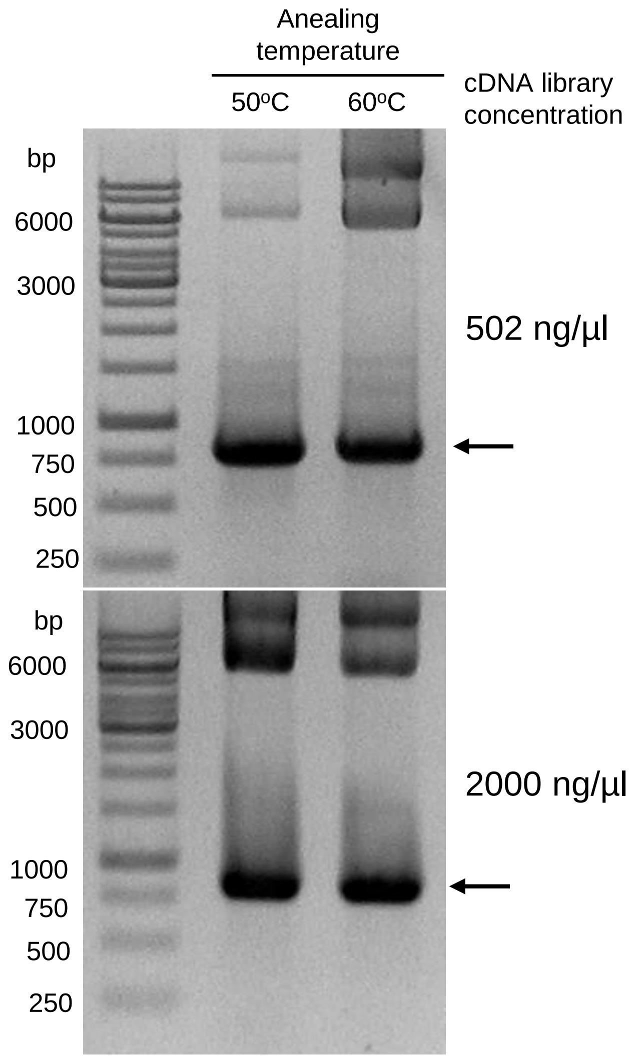

Supplementary Figure 1

Supplementary Figure 1 | Alternative splicing forms of *OsFER2*. **(A)** Alignment of the three alternative splicing forms of *OsFER2* cDNAs. The *OsFER2* cDNA sequences were collected from the Rice Genome Annotation Project (http://rice.uga.edu/). The arrows indicate the positons of primers to amplify the *OsFER2* gene. The alignment is presented by SnapGene Viewer version 5.2.4. **(B)** Percentage similarity of *OsFER2* cDNA sequences. The data were analyzed by Clustal2.1. **(C)** Amplification of *OsFER2* DNAs from rice cDNA library by *OsFER2*-Forward/Reverse (F/R) primers. The arrows indicates the OsFER2 band sizes.

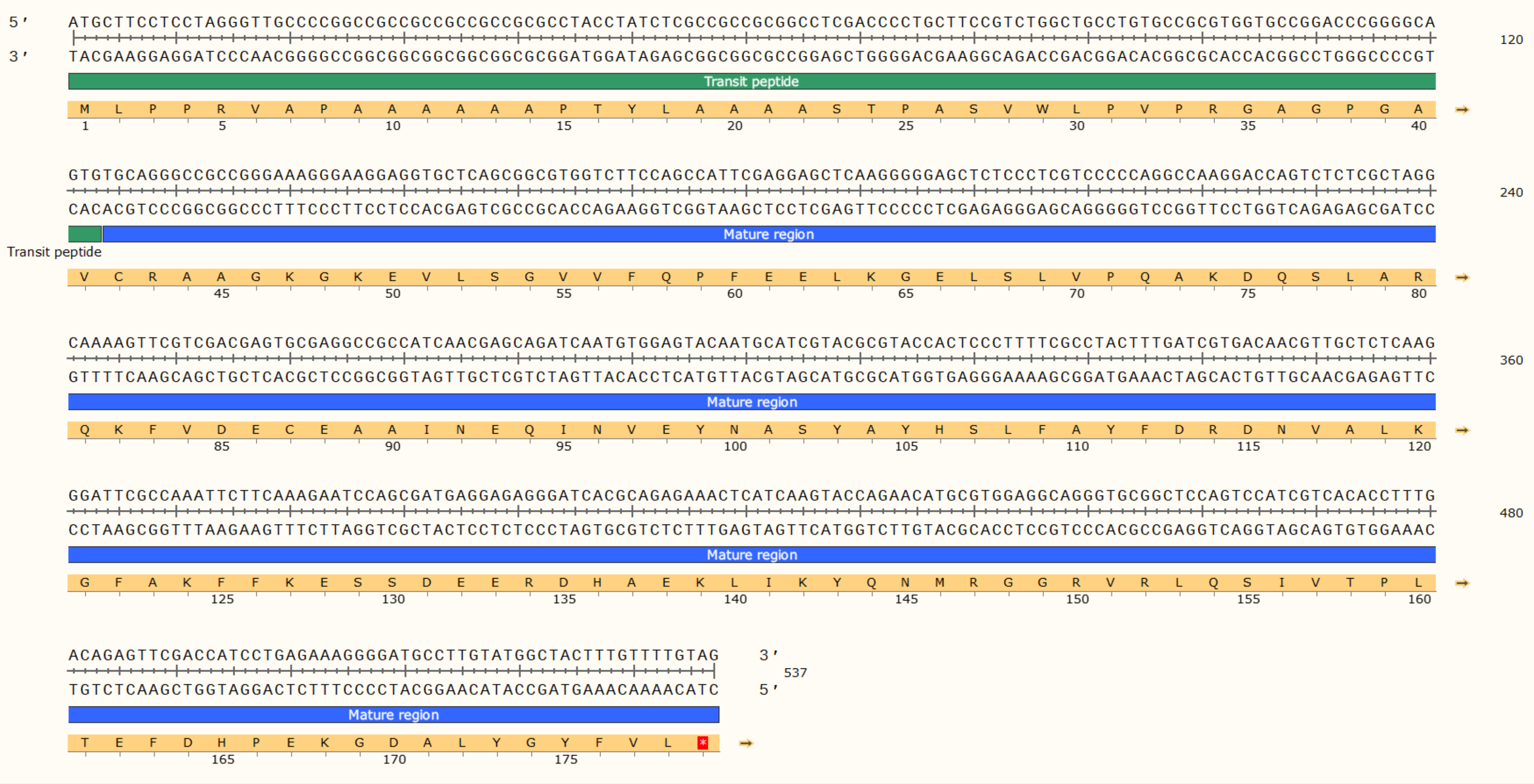

Supplementary Figure 2

Supplementary Figure 2. **|** The nucleotide sequence and deduced amino acid sequence of *OsFER1* cDNA. The *OsFer1* cDNA sequence is provided in SnapGene Viewer version 5.2.4. The amino acid sequences are indicated by a three-letter code below the respective nucleotide sequence. The OsFER1 protein is marked by the transit peptide (green) and mature region peptide (blue) bars below the nucleotide sequence. The arrows at the end of every line indicate the direction of translation. The asterisk (*) indicates the termination codon.

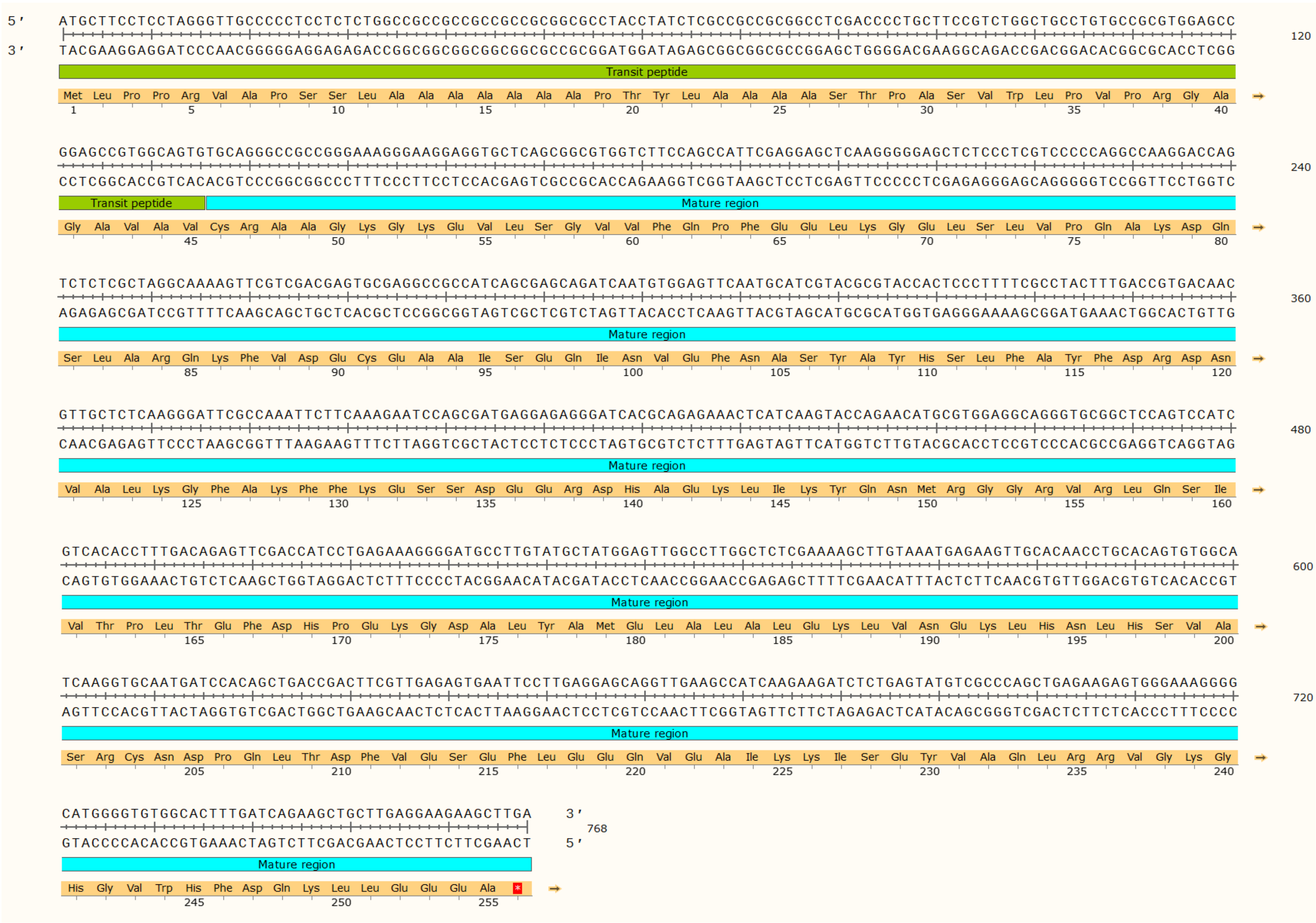

Supplementary Figure 3

Supplementary Figure 3. **|** The nucleotide sequence and deduced amino acid sequence of abundant *OsFER2* cDNA. The *OsFER2* cDNA sequence is provided in SnapGene Viewer version 5.2.4. The amino acid sequence is indicated by a three-letter code below the respective nucleotide sequence. The OsFER2 protein is marked by the transit peptide (green) and mature region peptide (blue) bars below the nucleotide sequence. The arrows at the end of every line indicate the direction of translation. The asterisk (*) indicates the termination codon.

	SFERH2	SFERH1	TritaFER2	ZmFER2	ZmFER1	TritaFER1	OsFER2	OsFER1	AtFER1	AtFER2	AtFER3	AtFER4	NtFER1	NtFER2	SFERH4	SFERH3
1: SFERH2	100.00	76.42	57.14	57.72	58.47	56.18	58.40	50.86	64.11	61.38	58.75	61.11	60.25	58.17	63.60	63.71
2: SFERH1	76.42	100.00	60.91	62.96	63.37	61.38	65.16	56.47	64.11	62.40	63.69	65.06	63.75	64.78	68.09	68.03
3: TritaFER2	57.14	60.91	100.00	75.20	77.24	80.32	77.73	72.94	58.94	59.76	59.38	61.85	61.83	60.48	61.98	65.18
4: ZmFER2	57.72	62.96	75.20	100.00	86.85	78.80	77.42	72.51	60.49	63.90	61.87	63.97	66.81	63.27	66.81	68.44
5: ZmFER1	58.47	63.37	77.24	86.85	100.00	82.54	81.60	75.72	60.82	63.79	63.13	63.05	65.69	64.63	66.10	67.76
6: TritaFER1	56.18	61.38	80.32	78.80	82.54	100.00	85.04	80.23	60.64	60.73	64.38	62.06	65.43	64.40	65.83	67.07
7: OsFER2	58.40	65.16	77.73	77.42	81.60	85.04	100.00	93.26	62.50	60.41	63.75	63.35	63.79	62.40	64.73	66.00
8: OsFER1	50.86	56.47	72.94	72.51	75.72	80.23	93.26	100.00	53.45	52.98	45.98	55.11	58.79	56.40	58.90	57.56
9: AtFER1	64.11	64.11	58.94	60.49	60.82	60.64	62.50	53.45	100.00	63.31	61.78	60.00	61.79	63.24	64.73	68.00
10: AtFER2	61.38	62.40	59.76	63.90	63.79	60.73	60.41	52.98	63.31	100.00	61.87	64.54	67.48	70.00	69.55	70.85
11: AtFER3	58.75	63.69	59.38	61.87	63.13	64.38	63.75	45.98	61.78	61.87	100.00	68.35	61.25	61.87	63.13	68.75
12: AtFER4	61.11	65.06	61.85	63.97	63.05	62.06	63.35	55.11	60.00	64.54	68.35	100.00	66.40	66.93	71.90	73.31
13: NtFER1	60.25	63.75	61.83	66.81	65.69	65.43	63.79	58.79	61.79	67.48	61.25	66.40	100.00	74.10	69.55	74.60
14: NtFER2	58.17	64.78	60.48	63.27	64.63	64.40	62.40	56.40	63.24	70.00	61.87	66.93	74.10	100.00	74.80	76.17
15: SFERH4	63.60	68.09	61.98	66.81	66.10	65.83	64.73	58.90	64.73	69.55	63.13	71.90	69.55	74.80	100.00	80.49
16: SFERH3	63.71	68.03	65.18	68.44	67.76	67.07	66.00	57.56	68.00	70.85	68.75	73.31	74.60	76.17	80.49	100.00

Supplementary Figure 4

Supplementary Figure 4. **|** Percentage similarity of amino acid sequences of OsFER and other plant ferritins. The percentage similarity was analyzed by Clustal2.1. Accession numbers: rice OsFER1 (Os11g01530), OsFER2 (Os12g01530), wheat TritaFER1 (AY864925), TritaFER2 (EU143671), maize ZmFER1 (X61391), ZmFER2 (X61392), soybean SFERH1 (M64337), SFERH2 (AB062754), SFERH3 (AB062755), SFERH4 (AB062755), AtFER1 (AT5G01600), AtFER2 (AT3G11050), AtFER3 (AT3G56090), AtFER4 (AT2G40300), tobacco NtFER1 (AY083924), and NtFER2 (AY141105).

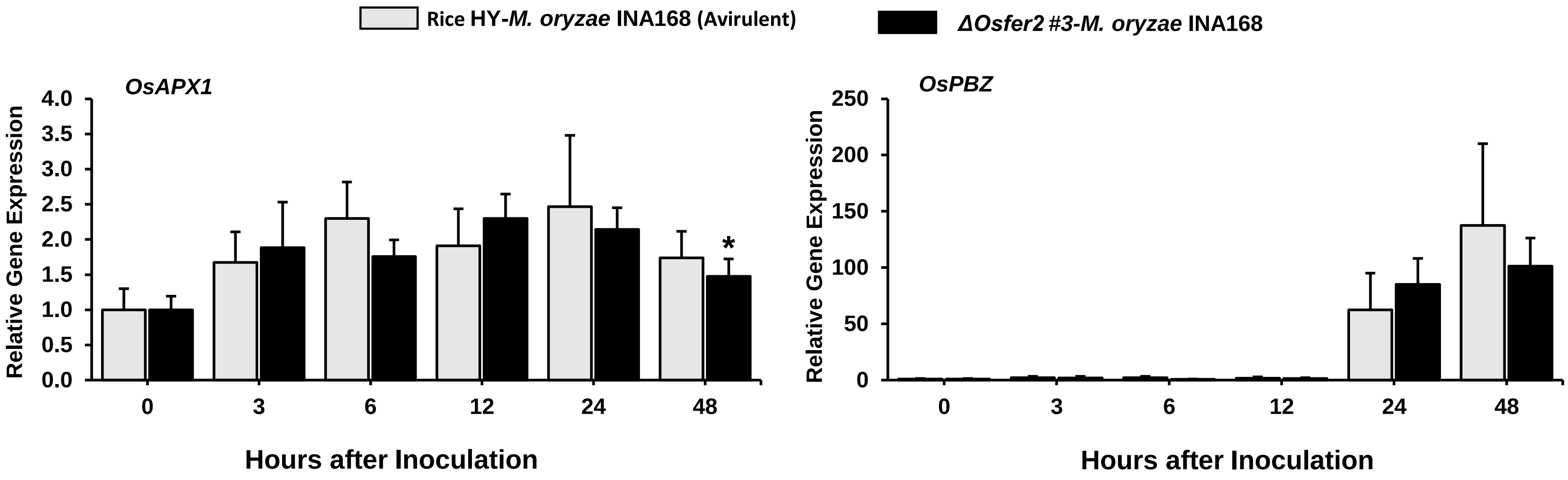

Supplementary Figure 5

Supplementary Figure 5 | Quantitative real-time RT-PCR analysis of time-course expression of *OsAPX1* and *OsPBZ1* in rice HY and Δ*Osfer2* mutant plants during avirulent *Magnaporthe oryzae* INA168 infection. Expression of ascorbate peroxidase1 (*OsAPX1*) and probenazole (PBZ)-induced protein1 (*OsPBZ1*) in rice leaf sheaths were analyzed by qRT-PCR. Relative expression levels at different time points during infection were calculated with the corresponding values at 0 hpi (control) after normalizing with respect to the expression of the internal control *OsUbiquitin*. Asterisks indicate statistically significant differences (Student's t-test, $^{*}P < 0.05$).

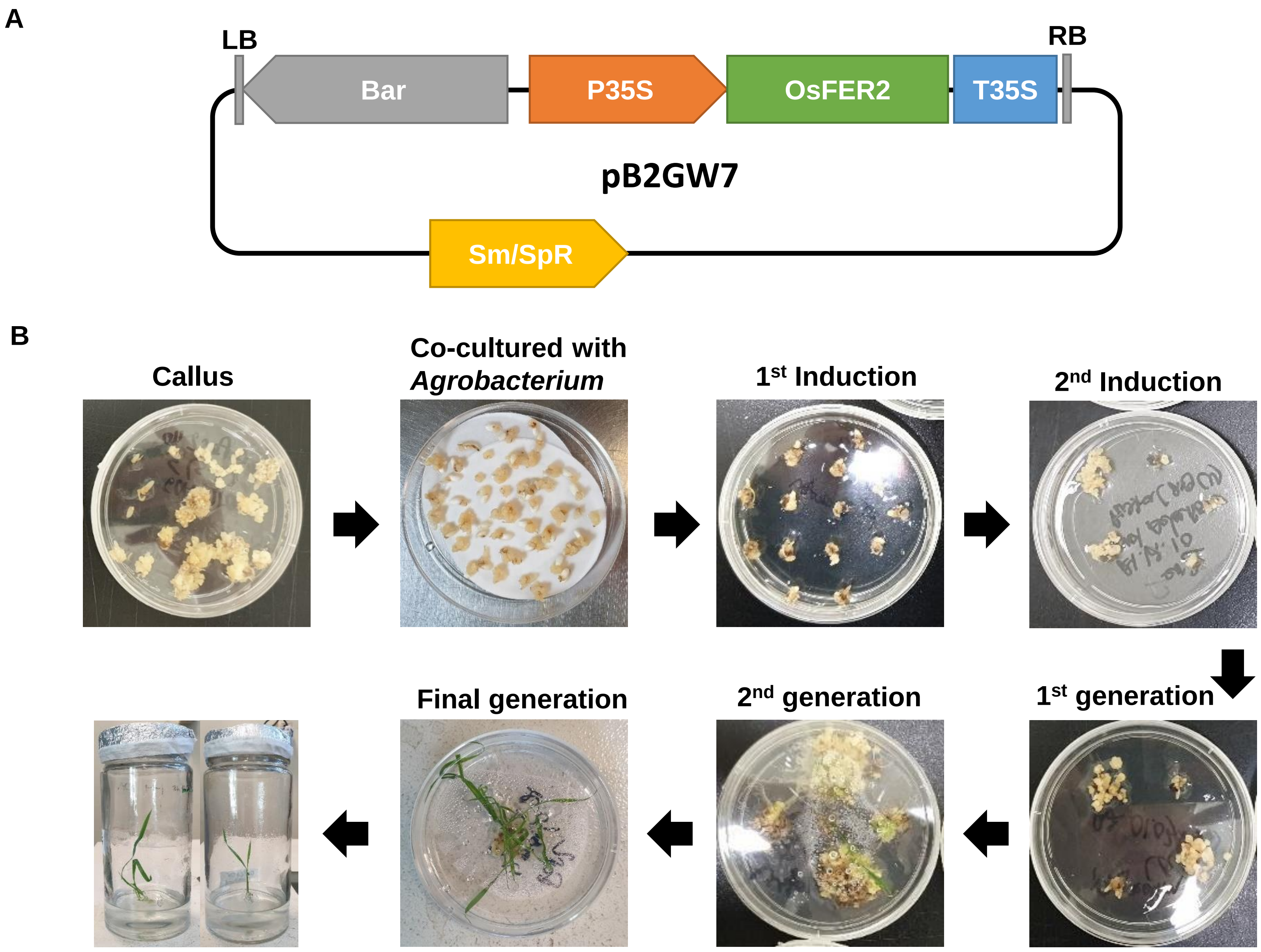

Supplementary Figure 6

Supplementary Figure 6 | Vector construction and flow chart for *OsFER2* complementation in *ΔOsfer2* #3 mutants. **(A)** Construction of the *OsFER2* complementation vector. The full-length *OsFER2* cDNA was cloned into the pB2GW7 vector carrying the CaMV 35S promoter. LB/RB, left/right T-DNA border; P35S/T35S, cauliflower mosaic virus (CaMV) 35S promoter/terminator; Bar, the coding region of the DL-phosphinothricin acetyltransferase gene that confers resistance to the herbicide glufosinate; Sm/SpR, Streptomycin/Spectinomycin bacterial Resistances. **(B)** Regeneration of *OsFER2* complementation plants through *Agrobacterium*-mediated transformation. The constructed CaMV 35S:*OsFER2* was transformed into *A. tumefaciens* strain LBA4404, and then transferred to *ΔOsfer2* #3 rice calli. Medium components and conditions for inducing transgenic rice calli and regenerating rice transformants are described in the Materials and Methods.

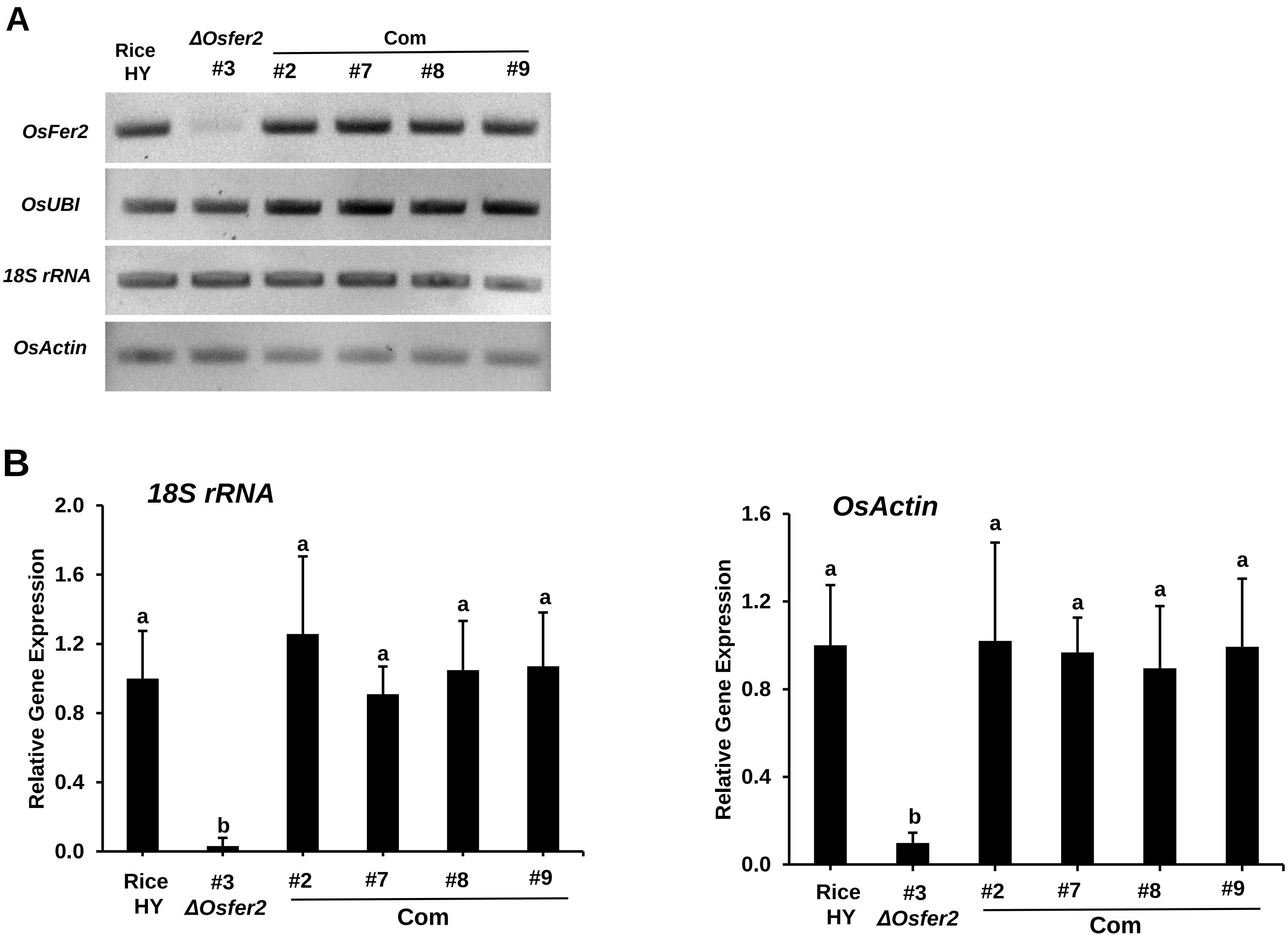

Supplementary Figure 7

Supplementary Figure 7 | (A) RT-PCR and **(B)** real-time qRT-PCR analyses of *OsFer2* expression in rice HY, *ΔOsfer2* #3 mutant and *OsFER2* complementation plants infected with avirulent *Magnaporthe oryzae* INA168. *OsFer2* expression in rice leaf sheaths was normalized using the expression of the internal control genes *OsUbiquitin, 18S rRNA and OsActin.* Asterisks indicate statistically significant differences, compared to wild-type rice HY plants (one-way ANOVA analysis, *$P<0.05$).

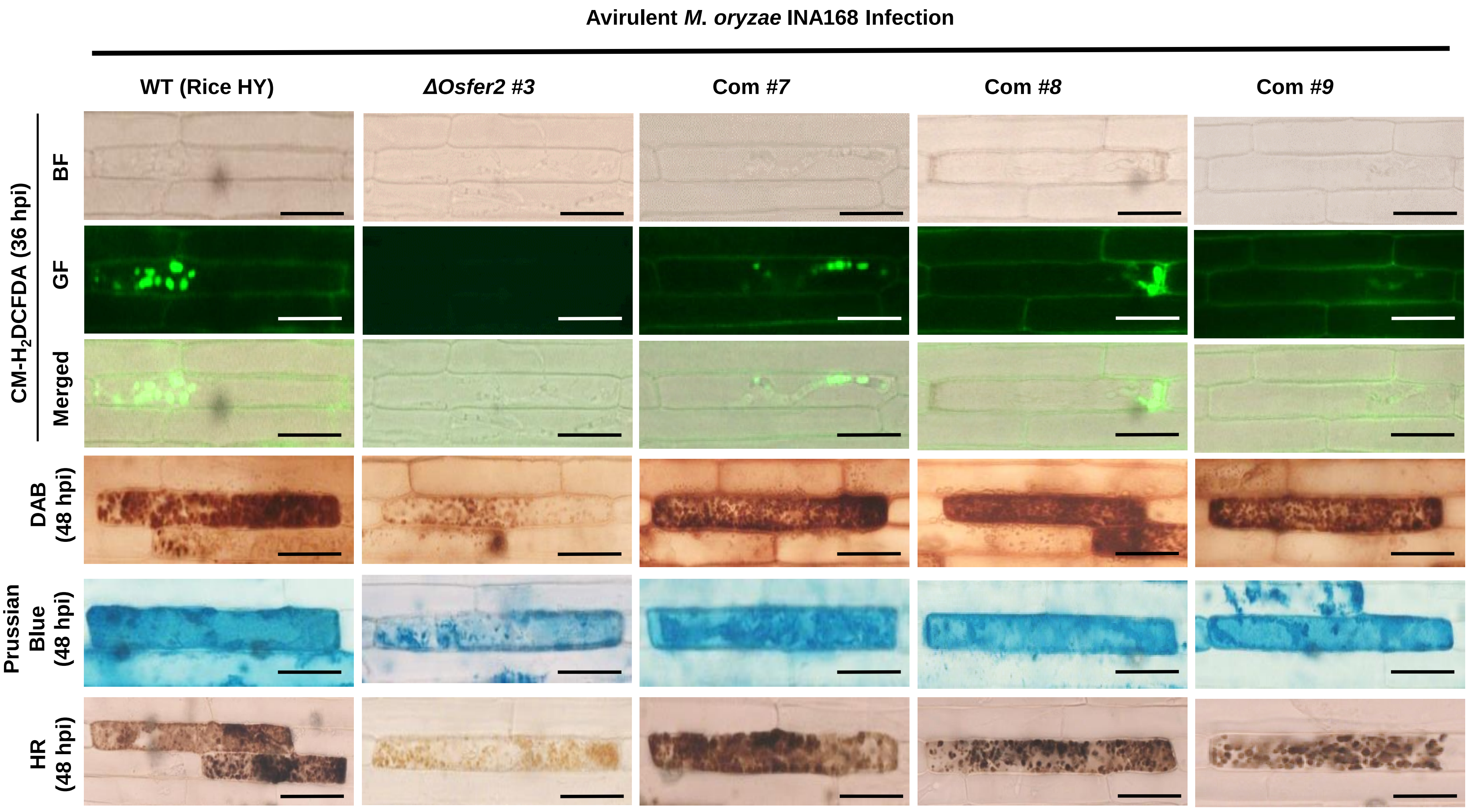

Supplementary Figure 8

Supplementary Figure 8 | Microscopic images of rice leaf sheath epidermal cells stained with CM-H_2DCFDA (green fluorescence), DAB and Prussian blue (Fe^{3+}, blue color) in different *OsFER2* complementation lines during avirulent *M. oryzae* INA168 infection. Images were captured using a fluorescence microscope (Zeiss equipped with Axioplan 2) with bright field and a combination of excitation (450–490 nm) and emission (515–565 nm) The images shown are representative of the different leaf sheath samples that were observed in three independent experiments. GF filters. hpi, hour post-inoculation; HR, hypersensitive response; BF, bright field; GF, green fluorescence. Scale bar=20 µm.
